# Interleukin‐12 sustained release system promotes hematopoietic recovery after radiation injury

**DOI:** 10.1002/mco2.704

**Published:** 2024-09-12

**Authors:** Chuanchuan Lin, Yang Xiang, Yangyang Zhang, Zhenxing Yang, Nanxi Chen, Weiwei Zhang, Lanyue Hu, Jianxin Chen, Ya Luo, Xueying Wang, Yanni Xiao, Qing Zhang, Xi Ran, Li Chen, Jigang Dai, Zhongjun Li, Qian Ran

**Affiliations:** ^1^ Laboratory of Radiation Biology, Department of Blood Transfusion, Laboratory Medicine Center The Second Affiliated Hospital, Army Medical University Chongqing China; ^2^ Hematopoietic Acute Radiation Syndrome Medical and Pharmaceutical Basic Research Innovation Center Ministry of Education of the People's Republic of China Chongqing China; ^3^ 111 Project Laboratory of Biomechanics and Tissue Repair, College of Bioengineering Chongqing University Chongqing China; ^4^ Institute of Respiratory Diseases The Second Affiliated Hospital, Army Medical University Chongqing China; ^5^ Department of Clinical Laboratory The Second Affiliated Hospital, Army Medical University Chongqing China; ^6^ Department of Thoracic Surgery The Second Affiliated Hospital, Army Medical University Chongqing China

**Keywords:** alginate hydrogel, bone marrow mesenchymal stem/stromal cells, hematopoietic injury and recovery, interleukin‐12, PI3K/AKT, radiation

## Abstract

The continuous production of mature blood cell lineages is maintained by hematopoietic stem cells but they are highly susceptible to damage by ionizing radiation (IR) that induces death. Thus, devising therapeutic strategies that can mitigate hematopoietic toxicity caused by IR would benefit acute radiation syndrome (ARS) victims and patients receiving radiotherapy. Herein, we describe the preparation of an injectable hydrogel formulation based on Arg‐Gly‐Asp‐alginate (RGD‐Alg) and Laponite using a simple mixing method that ensured a slow and sustained release of interleukin‐12 (IL‐12) (RGD‐Alg/Laponite@IL‐12). The local administration of RGD‐Alg/Laponite@IL‐12 increased survival rates and promoted the hematopoietic recovery of mice who had received sublethal‐dose irradiation. Local intra‐bone marrow (intra‐BM) injection of RGD‐Alg/Laponite@IL‐12 hydrogel effectively stimulated IL12 receptor‐phosphoinositide 3‐kinase/protein kinase B (IL‐12R‐PI3K/AKT) signaling axis, which promoted proliferation and hematopoietic growth factors secretion of BM mesenchymal stem/stromal cells. This signaling axis facilitates the repair of the hematopoietic microenvironment and plays a pivotal role in hematopoietic reconstitution. In conclusion, we describe a biomaterial‐sustained release of IL‐12 for the treatment of irradiated hematopoietic injury and provide a new therapeutic strategy for hematopoietic ARS.

## Graphical abstract

Schematic illustration of the mechanism of Arg‐Gly‐Asp‐alginate (RGD‐Alg)/Laponite@IL‐12 hydrogel as an effective therapeutic strategy to treat radiation‐induced hematopoietic injury.

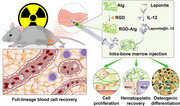

## 1 INTRODUCTION

The escalating threat of nuclear terrorism, coupled with the swift progress of nuclear energy and medicine, underscores the pressing need to devise efficient medical countermeasures for radiation exposure. Acute radiation syndrome (ARS) arises from exposure to high doses of ionizing radiation (IR) even within a brief timeframe. Poor hematopoietic recovery has been reported to be the main cause of death in IR exposure dose of 10 Gy.^1^ Due to the high intrinsic replication rate, the hematopoietic system exhibits particular radiosensitivity, making hematopoietic ARS (H‐ARS) a prevalent condition.^2^ H‐ARS typically manifests as myelosuppression, immunosuppression, thrombocytopenia, and anemia, resulting from the IR damage to hematopoietic stem cells (HSCs) and/or hematopoietic microenvironment.^3^ Despite the growing understanding of the importance of early intervention in the prevention of clinical consequences after IR, few therapeutic radioprotectants for H‐ARS have been developed.

Treatment of H‐ARS includes blood transfusion, hematopoietic cytokines treatment, and HSC transplants (HSCT). In recent years, hematopoietic cytokines have received great attention as an effective treatment for H‐ARS. Currently, the US Federal Drug Administration (FDA) has approved three hematopoietic cytokines, namely granulocyte colony‐stimulating factor (G‐CSF)/Neupogen, PEGylated G‐CSF/Neulasta, and recombinant human granulocyte‐macrophage colony‐stimulating factor (GM‐CSF)/Leukine, for the treatment of H‐ARS.^4^ Unfortunately, these cytokines can only stimulate the recovery of a certain lineage or a few lineages of blood cells, and accelerate the exhaustion of survived HSCs.^5^, ^6^ Allogeneic HSCT is a life‐saving and curative treatment in patients with hematologic malignant neoplasms. However, the use of HSCT in H‐ARS patients with bone marrow (BM) failure remains controversial, with evidence suggesting that the effect on survival is unclear.^7^ Meanwhile, HSCT is not always available following a mass casualty radiological event.^8^ Some aminothiol drugs have also been proposed for mitigation of IR‐induced hematopoietic injury. Currently, only amifostine has been clinically approved by the US FDA for mitigating the impact of IR, but its clinical application is limited due to serious adverse reactions.^9^ Nevertheless, these therapies cannot achieve long‐term hematopoietic reconstitution to overcome the radiation damage to the hematopoietic microenvironment.^10^, ^11^ The maintenance of hematopoietic functions depends not only on HSCs but also on the supportive role of the hematopoietic microenvironment.^12^ The hematopoietic microenvironment is indispensable for self‐renewal, proliferation, and differentiation of HSCs, a process facilitated by direct cell‐cell contact or soluble chemical factors. BM‐MSCs play a pivotal role in the regulation of HSCs, and the enhancement of osteogenic differentiation in BM‐MSCs is also involved in maintaining HSCs function.^13^ Thus, mesenchymal stem/stromal cell (MSC)‐based therapeutic strategies are also an option to promote hematopoietic recovery following acute radiation injury.^14^ Although there are numerous studies describing the effects of hematopoietic stimulation of MSCs in preclinical rodent models of ARS, evidence supporting consistent cell engraftment is still lacking, and further studies on the underlying mechanisms are necessary.^15^, ^16^ Therefore, therapeutic strategies to repair HSCs and the hematopoietic microenvironment may be more promising.

An optimal radiation medical countermeasure would be able to reconstitute all blood cell lineages and demonstrate effectiveness within hours to days after IR exposure, ideally with a single dose, given the lack of comprehensive supportive care in widespread radiological emergencies.^17^ Interleukin‐12 (IL‐12) is a heterodimeric pro‐inflammatory cytokine that plays an important role in the innate and adaptive immune systems; it regulates the inflammatory response, helps natural resistance to infection, and participates in the adaptive immune response.^18^ Accumulated studies have documented that a single administration of IL‐12 can improve survival in murine and rhesus monkey models after lethal irradiation.^5^, ^17^, ^19^ Different from most hematopoietic cytokines, IL‐12 can effectively stimulate BM regeneration and increase nadir levels of all major blood cell types, including lymphocytes, neutrophils, red blood cells, reticulocytes, and platelets.^20^ However, IL‐12 also produces undesirable severe side effects, such as fatigue, dyspnea, stomatitis, acidosis, gastrointestinal hemorrhage, and even death,^21^ which limit its potential use for the treatment of H‐ARS. Therefore, there is an unmet need for successful strategies that minimize or eliminate the toxicity of IL‐12 while locally improving its hematopoietic activation. Thus, there is considerable interest in the development of IL‐12 delivery systems that include gene delivery strategies,^22^ fusion or recombinant protein,^23^ and controlled‐release polymers.^24^, ^25^ Furthermore, safe methods to locally and sustainably release IL‐12 in the BM and accelerate hematopoietic reconstitution remain an urgent challenge.

Compared to systemic delivery, macroscopic protein drug delivery systems offer a means of controlling drug administration to specific tissues, thus minimizing side effects and improving the efficacy of treatment at the intended target site.^26^, ^27^ Hydrogels are water‐swellable networks of cross‐linked hydrophilic polymers and have been effectively utilized as matrices to protect proteins and allow their controlled release at targeted tissue locations.^28^ However, the majority of hydrogels possess mesh sizes that exceed the average protein size, resulting in weak interactions with the polymer backbone of the hydrogel, which in turn allows fast diffusion‐based release from these matrices. For instance, alginate (Alg), a polysaccharide commonly used in the delivery of, can efficiently encapsulate proteins under mild conditions by crosslinking with calcium ions^29^; however, Alg polymers do not form strong interactions with numerous proteins, resulting in the rapid release of proteins from Alg hydrogels.^27^, ^30^ The controlled release of proteins from hydrogels can be achieved by incorporating particles that strongly interact with proteins within the hydrogel matrix. Laponite, a synthetic smectite clay nanoplatelet with a high negative surface charge, is a potential particle for use in controlled protein release from hydrogels.^31^ Previous research has demonstrated the efficacy of Laponite as a carrier for small‐molecule drugs.^32^, ^33^ As a result, it is commonly incorporated into various hydrogel systems to create nanocomposite gels for controlling protein release.

Herein, we described the development of a nanocomposite hydrogel system based on Alg and Laponite. By pre‐absorbing IL‐12 onto charged Laponite nanoplatelets and incorporating them into injectable hydrogels, we proposed it would be possible to achieve sustained release of a bioactive protein that stimulates hematopoietic recovery. Furthermore, Laponite is easily degraded in physiological environments, resulting in non‐toxic and even bioactive products.^27^, ^30^ Recent studies have reported the excellent osteo‐inductive effect of Laponite in MSCs and osteoblasts,^34^, ^35^, ^36^ which is beneficial in maintaining hematopoietic function.^37^ The polypeptide Arg‐Gly‐Asp (RGD) was grafted onto Alg to provide a cell adhesion site for cell growth and tissue regeneration. Intraosseous BM injection (IBMI) is often used for stem cell and hematopoietic cell transplants.^10^ To achieve sustained localized release of IL‐12, IBMI was used for hydrogel delivery.

We expected that the fabricated RGD‐Alg/Laponite@IL‐12 hydrogel would be retained in the BM to locally release cytokines. The Hydrogel system could repair hematopoietic damage after irradiation. Further, the degraded hydrogel products could effectively promote osteogenic differentiation of BM‐MSCs in the BM microenvironment, thereby benefiting long‐term hematopoietic recovery. Ultimately, ideal hematopoietic repair will be achieved.

## 2 RESULTS

### 2.1 Physicochemical characterization and IL‐12 release properties of the RGD‐Alg/Laponite@IL‐12 hydrogel

**FIGURE 1:**
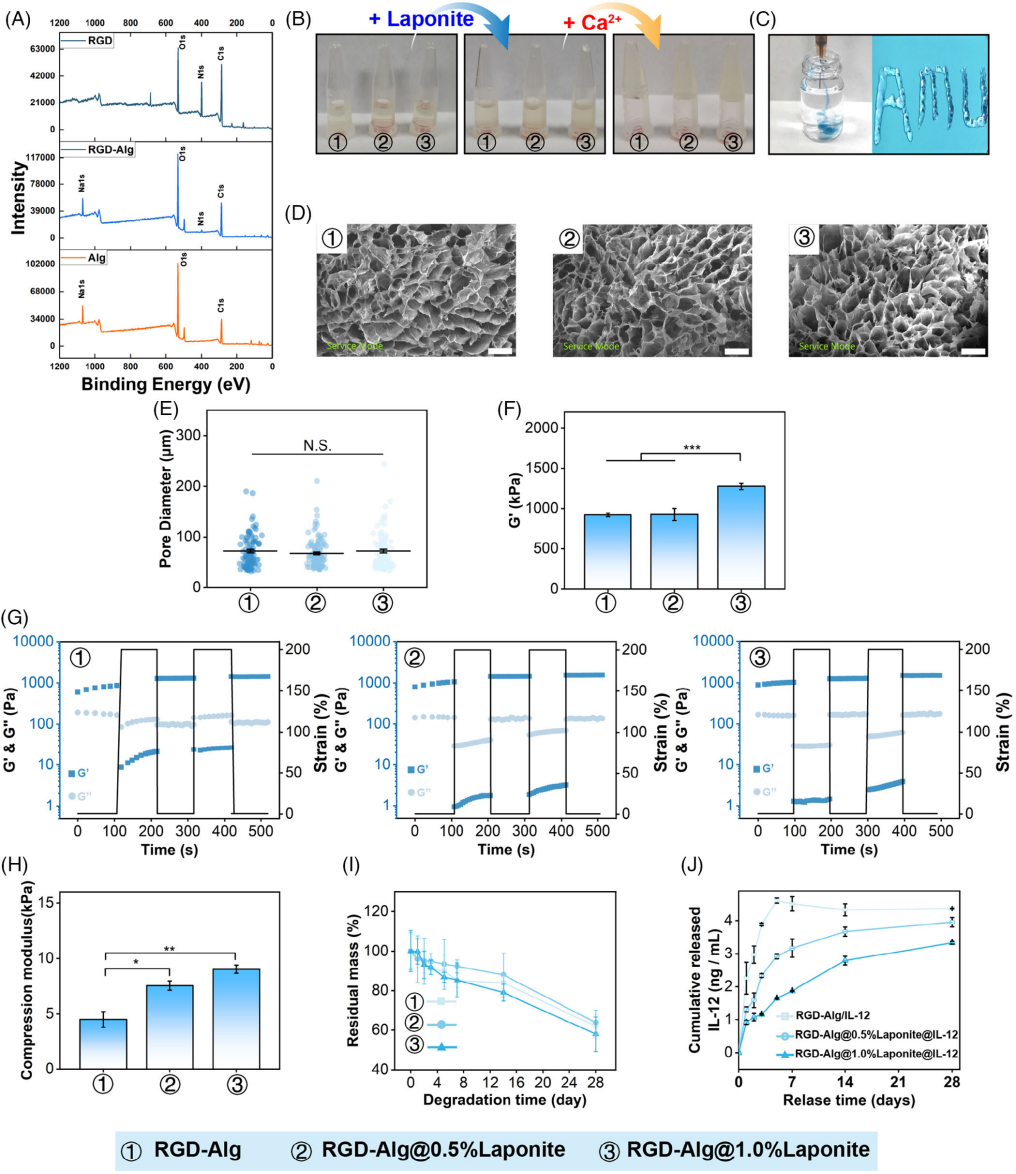
(A) X‐ray photoelectron spectroscopy (XPS) survey spectra of pure sodium alginate (Alg), pure GGGGRGDSP peptide (RGD), and sodium alginate grafted GGGGRGDSP (RGD‐Alg); (B) photographs of hydrogel gelation testing; (C) photographs of hydrogel injected via a fine needle; (D, E) scanning electron microscopy (SEM) images and pore diameter of lyophilized hydrogel (scale bar = 100 µm); (F) equilibrium storage modulus of the hydrogels (*n* = 3); (G) four‐cycle step‐strain measurement with alternate step strains of 0.5% and 200% at fixed angular frequency of 10 rad/s; (H) compression modulus of hydrogels (*n* = 3); (I) degradation of hydrogel in phosphate‐buffered saline (PBS) solution (*n* = 5); (J) IL‐12 release profiles of different hydrogels (RGD‐Alg/IL‐12, RGD‐Alg/0.5% Laponite@IL‐12, RGD‐Alg/1.0% Laponite@IL‐12) (*n* = 3).

Alginate‐based hydrogels for drug delivery applications have received growing attention because of their ability to enable site‐specific drug delivery while avoiding potential side effects. Despite the excellent properties of biocompatibility and hydrophilicity, the lack of a cell adhesion domain reduces the drug delivery applications. In this study, synthetic peptides containing RGD were covalently grafted with Alg to promote cell adhesion and improve histocompatibility.^38^ As shown in Figure 1A, the N1s signal centered at 400 eV was missing in pure Alg and only appeared in the spectra of RGD and RGD‐Alg, suggesting that the N1s signal observed in RGD‐Alg originates from the successful grafting of RGD onto pure Alg. The X‐ray photoelectron spectroscopy (XPS) data show that RGD‐Alg was successfully synthesized.

Due to the enrichment of structures of guluronic acid, the Alg solution can create ionic crosslinking networks in the presence of divalent cations such as Ca^2+^.^38^ The ionic interaction between the Ca^2+^ and Alg was the driving force for forming shear‐thinning hydrogels.^39^ As shown in Figure 1B, the hydrogel precursor solutions can only form a gel in the presence of Ca^2+^ and injected through a 26G syringe needle (Figure 1C). According to scanning electron microscopy (SEM) images, different concentrations of Laponite did not obviously alter the micropores structure of RGD‐Alg/Laponite@IL‐12 hydrogel (Figure 1D), which showed a typical continuous structure with ∼70 µm of interconnected micropores (Figure 1E). This internal structure facilitates cell growth invasion and tissue repair.^40^

The mechanical properties of hydrogels were measured by rheology and compression tests. In our study, the storage modulus increased with increasing Laponite concentration (Figure 1F), and the G’ of the hydrogel was higher than G’’ in the frequency range (Figure 1G), which indicated that the RGD‐Alg hydrogel was viscoelastic. Furthermore, increasing concentrations of Laponite also promoted the hydrogel compression modulus (Figure 1H), with the highest compressive strength of the RGD‐Alg/Laponite@IL‐12 hydrogel being ∼9 kPa.

The degradation curve of the pure RGD‐Alg and RGD‐Alg/Laponite hydrogel did not show an apparent difference (Figure 1I). All gels degraded by ∼60% after 28 days. As shown in Figure 1J, Laponite effectively slowed the IL‐12 release rate. The cumulative release of IL‐12 from pure RGD‐Alg reached a plateau at approximately 5 ng/mL on day 5. However, no discernible plateaus were observed in the RGD‐Alg/Laponite gels. After a 14‐day period, the RGD‐Alg/0.5% Laponite gel had released approximately 4 ng/mL of IL‐12, while the RGD‐Alg/1% Laponite group released approximately 3 ng/mL at the same time. Consequently, in our subsequent research, the RGD‐Alg/1% Laponite gel was chosen as the slow‐release carrier for IL‐12.

### 2.2 Biocompatibility evaluation of the RGD‐Alg/Laponite@IL‐12 hydrogel

**FIGURE 2:**
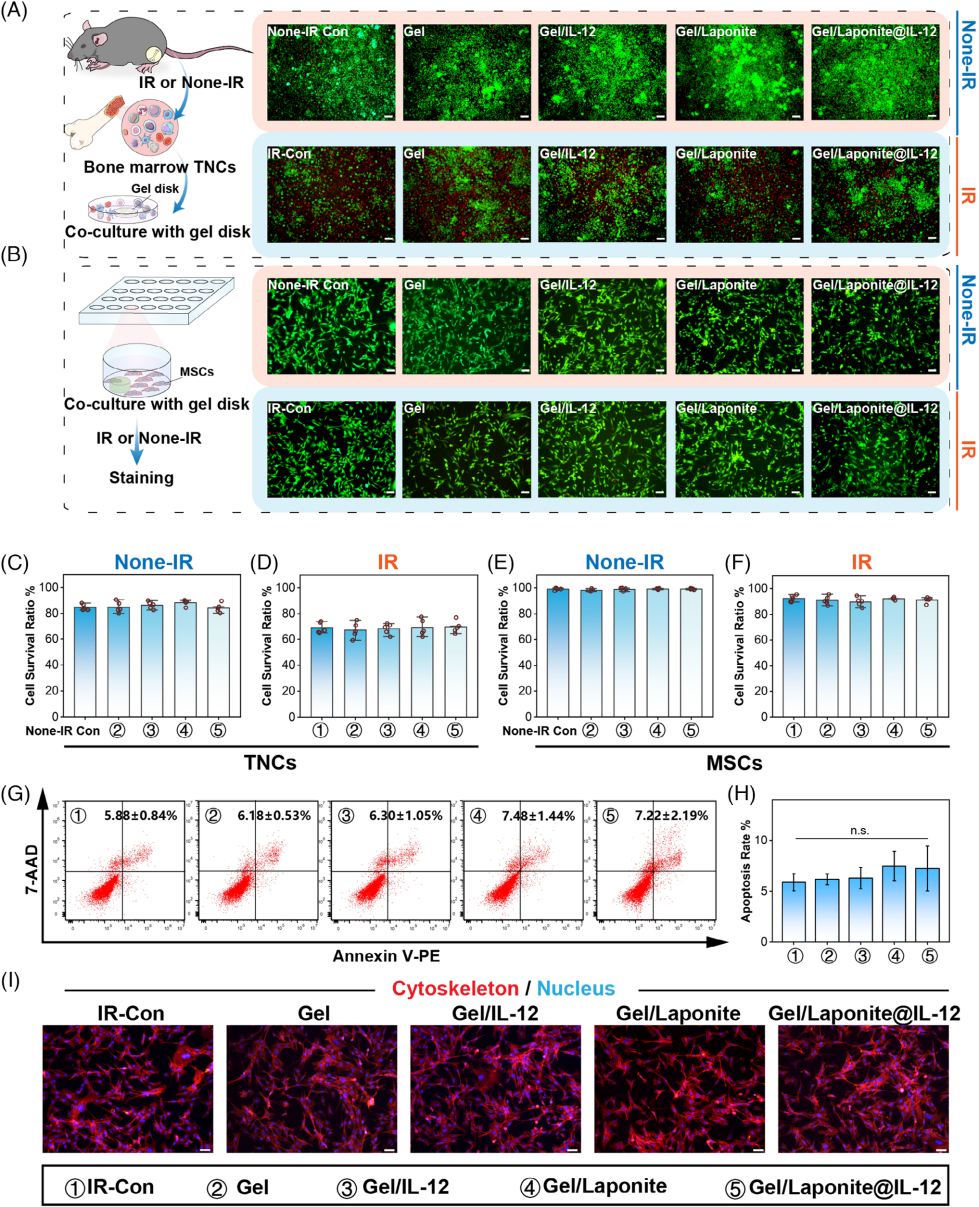
Characterization of hydrogel biocompatibility in vitro. The effects of hydrogel on the survival of bone marrow (BM) total nucleated cells (TNCs) (A) and bone marrow‐mesenchymal stem/stromal cells (BM‐MSCs) (B) under irradiated (IR) or none‐irradiated (None‐IR) conditions; the effects of hydrogel on the cell survival ratio of TNCs (C, D) and BM‐MSCs (E, F) under IR or None‐IR conditions (*n* = 5); (G, H) the effects of hydrogel on the apoptosis of BM‐MSCs (*n* = 3); (I) images of the morphological changes of BM‐MSCs subsequent to the hydrogel under IR conditions (scale bar = 100 µm).

To verify the biocompatibility of the hydrogel, BM total nucleated cells (TNCs) and BM‐MSCs were adopted in this paper. As shown in Figure 2A, live/dead assays of BM TNCs were conducted using fluorescein diacetate (FDA)/propidium iodide (PI) staining. The staining results showed no sign of cytotoxicity of Gel, Gel/IL‐12, Gel/Laponite, and Gel/Lapnite@IL‐12. Similarly, the viability staining evaluation of BM‐MSCs did not reveal any notable disparities among the various groups (Figure 2B). Cell morphology, differentiation assays, and surface markers were used to identify the BM‐MSCs (Figure S1). The identification of BM‐MSCs is presented in Figure S1. The results of the survival ratio assay confirmed that the cell viability of BM TNCs (Figure 2C,D) and BM‐MSCs (Figure 2E and Figure 2F) did not change significantly due to the addition of hydrogel under irradiation (Figure 2D,F) or not (Figure 2C,E). Furthermore, the apoptosis and morphology of BM‐MSCs in each group also did not exhibit marked differences (Figure 2G–I). To assess their potential for biological applications, the in vivo biocompatibility of the Gels was evaluated. At 7 days after irradiation and implantation, no evident tissue damage or inflammatory lesions were detected in the main organs compared to the organs of the control group (Figure S2). Given these observations, it can be concluded that the IL‐12 sustained‐release system used in our study exhibited good biocompatibility.

### 2.3 RGD‐Alg/Laponite@IL‐12 hydrogel promoted survival and hematopoietic recovery after irradiation

**FIGURE 3:**
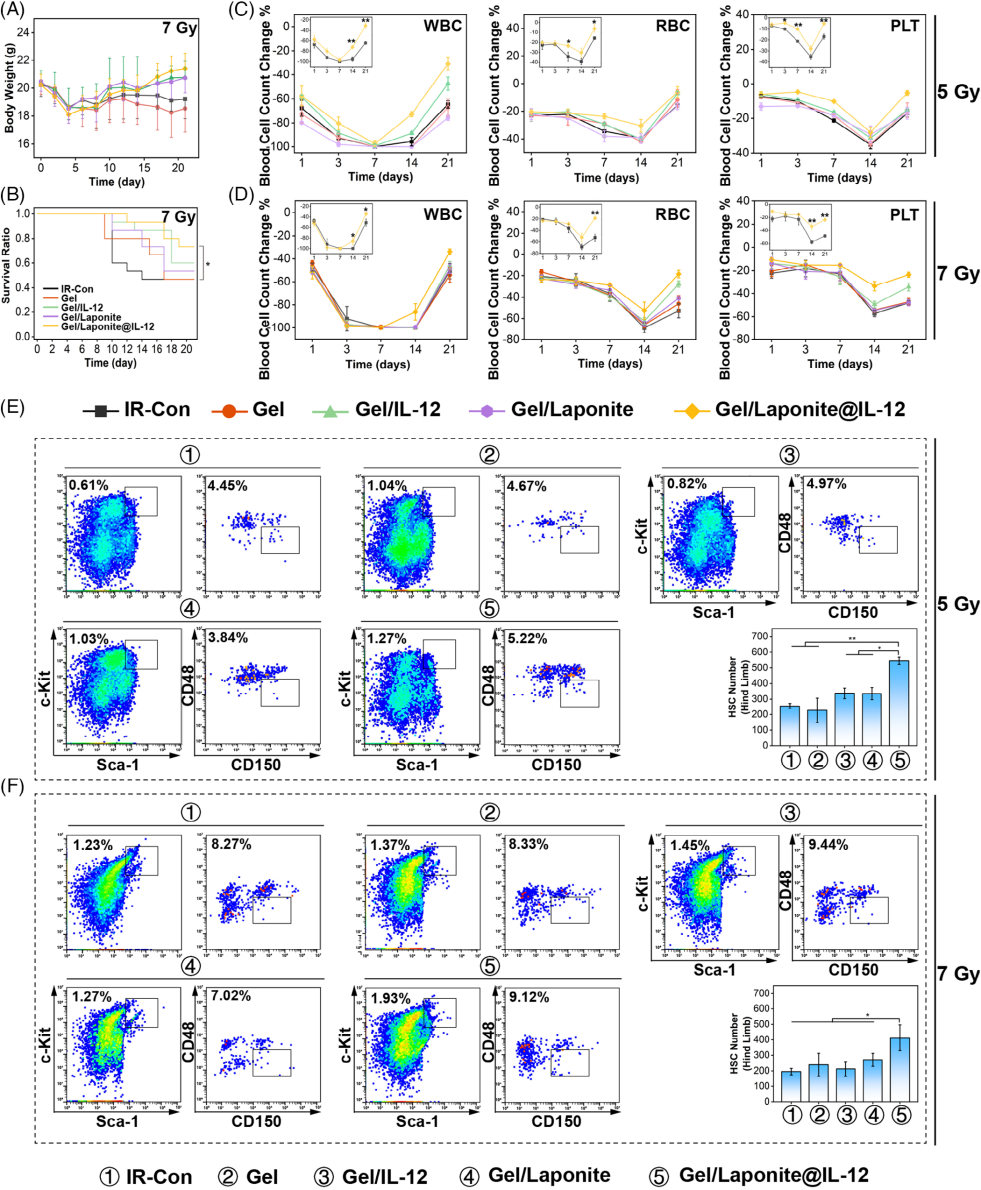
Hydrogel‐enhanced hematopoietic stem cells (HSCs) regeneration after total body irradiation (TBI). (A) Body weights of mice with different treatments after receiving the 7 Gy irradiated dose; (B) the effects of different treatments on the survival ratio of mice after sublethal‐dose irradiation (7 Gy); Gel/Laponite@IL‐12 promoted the recovery of peripheral blood cell count (PBCC) after 5 Gy irradiation (C) and attenuated the decline of PBCC after 7 Gy irradiation (D); The percentages and the numbers of HSCs in the bone marrow of 5 Gy (I) or 7 Gy (J) irradiated mice on day 7 after receiving different treatments.

To evaluate the protective effects of the IL‐12 sustained‐release hydrogel system on mice exposed to irradiation, body weight, survival, and hematopoietic recovery were measured after irradiation with 5 Gy or 7 Gy (sublethal‐dose). The animal received intraosseous injections of normal saline (IR‐Con) and different hydrogels (Gel, Gel/IL‐12, Gel/Laponite, Gel/Laponite@ IL‐12) rapidly within 1 h after irradiation. Following exposure to 5 Gy irradiation, no distinct differences in body weight percentage among the groups were observed (Figure S3A). However, sublethal‐dose irradiated mice that received Gel/Laponite@IL‐12 hydrogel showed obviously less body weight loss compared to animals that received normal saline (Figure 3A). Furthermore, the survival ratio increased significantly in the Gel/Laponite@IL‐12 group (Figure 3B), with a significant difference observed between the IR‐Con and the Gel/Laponite@IL‐12 treated group.

The peripheral blood cell count (PBCC) and BM pathological morphology were also investigated. The results presented in Figure 3C,D indicated that compared to the IR‐Con group, the Gel/Laponite@IL‐12 treatment effectively prevented peripheral blood cell decrease to nadir and promoted the recovery of PBCC in irradiated mice. In Figure S4, the BM structure and cellularity of the irradiated mice displayed severe damage on day 7, however, mice treated with Gel/Laponite@IL‐12 showed noticeably higher levels of TNC in their femurs. Lastly, to assess the impact of this gel on the restoration of functional HSCs, BM TNCs were isolated and analyzed by flow cytometry (Figure S3B). As shown in Figure 3E, on day 7 following the 5 Gy irradiation, mice treated with Gel/Laponite@IL‐12 displayed increased numbers of HSCs compared to the IR‐Con. Similarly, after sublethal‐dose irradiation, there was also a greater number of HSCs in Gel/Laponite@IL‐12‐treated mice (Figure 3F). The increased number of HSCs indicated that Gel/Laponite@IL‐12 protects from irradiation damage by promoting the recovery of hematopoiesis. In addition, after 7 Gy irradiation, there was no significant difference in survival (Figure S3D) or weight change (Figure S3E) between mice treated with IL‐12 and Gel/IL‐12. Similar results were also observed in the recovery of PBCC (Figure S3F) and HSCs (Figure S3G,H).

### 2.4 Gel/Laponite@IL‐12 hydrogel accelerated hematopoietic microenvironment repair by promoting the proliferation, secretion, and osteogenic differentiation of BM‐MSCs

**FIGURE 4:**
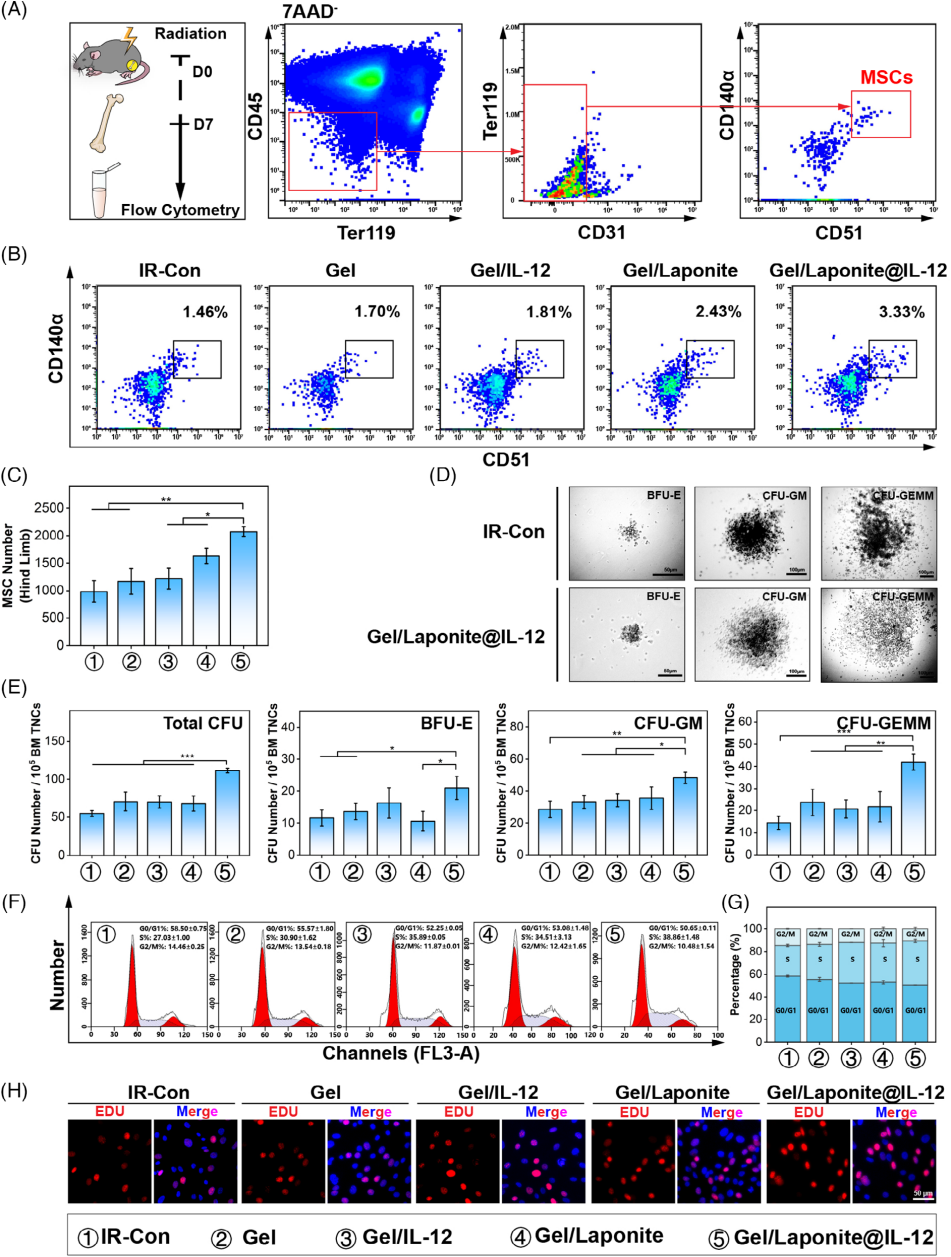
Hydrogel increased the number and secretion function of bone marrow‐mesenchymal stem/stromal cells (BM‐MSCs). (A) Schematic diagram of the experimental process and representative flow cytometry analysis of BM‐MSCs; (B, C) percentages and number of BM‐MSCs in the bone marrow of irradiated mice on day 7 after receiving different treatment (*n* = 3); (D, E) analysis of the hematopoietic colony formation ability of the TNCs after co‐culture with BM‐MSCs which receiving different treatments; (F, G) the cell cycle analysis of BM‐MSCs was conducted 48 h after receiving various treatments (*n* = 3); (H) displays representative images of EdU‐positive cells in BM‐MSCs under different conditions after 48 h (scale bar = 50 µm).

Given the crucial role of BM‐MSCs in hematopoietic reconstitution after exposure to irradiation, we therefore asked whether Gel/Laponite@IL‐12 had effects on BM‐MSCs. We first evaluated the change in the number of BM‐MSCs in the BM of irradiated mice that received different treatments (Figure 4A). According to flow cytometric analysis, no significant differences were observed in the percentages of BM‐MSCs between BM samples from the IR‐Con, Gel, and Gel/IL‐12 groups. However, there was a significantly increased number of BM‐MSCs in Gel/Laponite and Gel/Laponite@IL‐12‐treated mice (Figure 4B,C).

The main mechanism of radiation protective activity of BM‐MSCs involves secretory effects to promote hematopoietic reconstitution.^8^ Subsequently, we investigated the impact of various treatments on the secretion function and proliferation of BM‐MSCs in vitro. Figure 4D,E shows the total number of BM‐derived colony‐forming units (CFUs), specifically burst‐forming unit‐erythroid (BFU‐E), CFU‐granulocyte and macrophage (CFU‐GM), and CFU‐granulocyte, erythrocyte, monocyte, megakaryocyte (CFU‐GEMM), notably increased in the group co‐cultured with Gel/Laponite@IL‐12‐treated BM‐MSCs compared with the IR‐Con group. Thus, Gel/Laponite@IL‐12 treatment may promote hematopoietic recovery after irradiation by improving the secretory function of BM‐MSCs. The cell cycle assay revealed that Gel/Laponite@IL‐12 treatment caused more BM‐MSCs to enter the S phase. Conversely, IR‐con treatment led to greater G0/G1 phase arrest (Figure 4F,G). Consistent with cell cycle analysis, the Gel/Laponite@IL‐12‐treated BM‐MSCs expressed more EdU‐positive cells compared to the IR‐Con group (Figure 4H). These cell proliferation‐related results indicated that Gel/Laponite@IL‐12 treatment could effectively promote the proliferation of BM‐MSCs in vitro and in vivo after irradiation, which was conducive to the recovery of the hematopoiesis.

**FIGURE 5:**
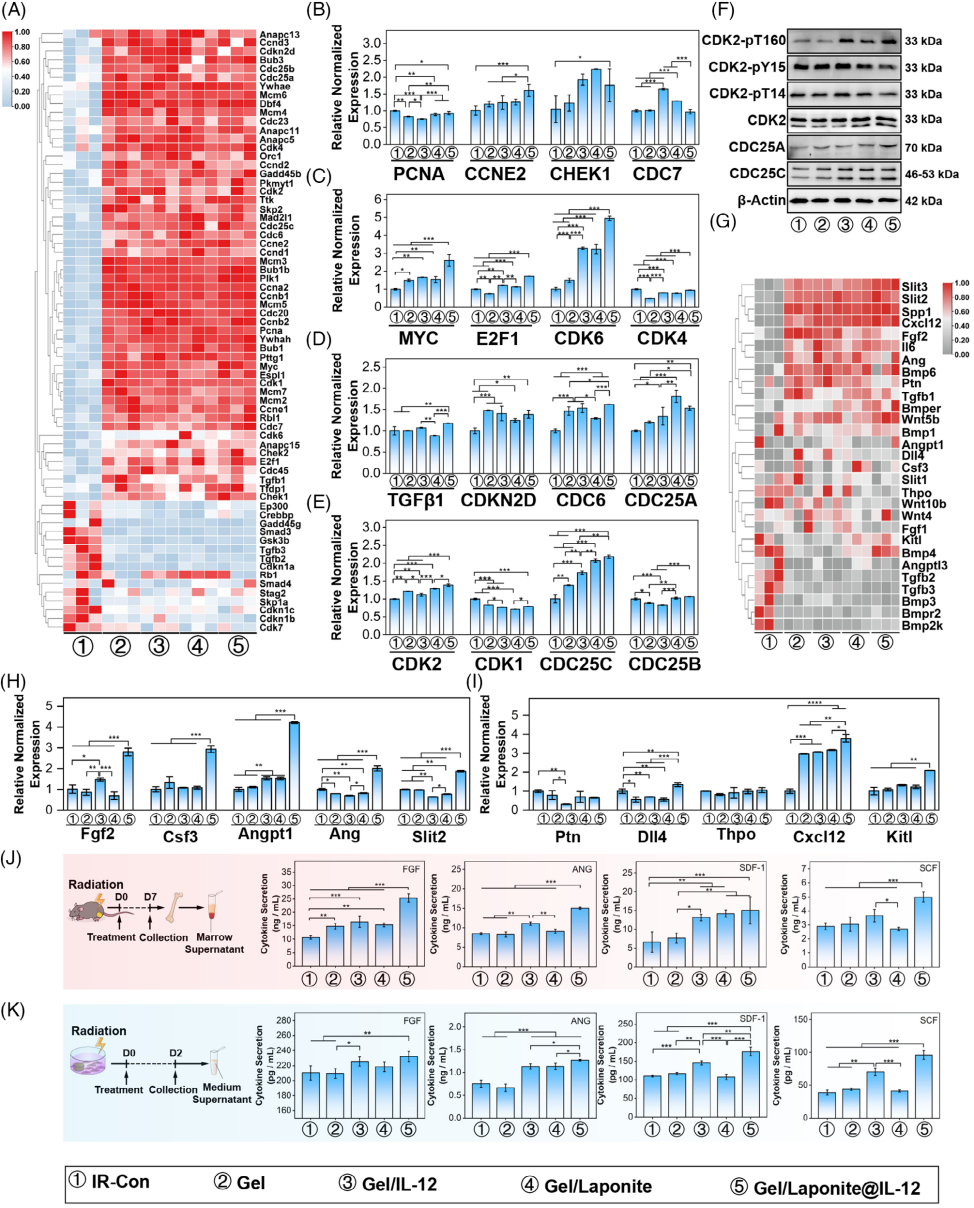
(A) Heatmap showing expression of cell cycle‐related genes. (B–E) Expression changes of cell cycle‐related genes in bone marrow‐mesenchymal stem/stromal cells (BM‐MSCs) with different treatments (*n* = 3). (F) Expression of cell cycle‐related proteins in BM‐MSCs after receiving different treatment. (G) Heatmap showing the expression of hematopoietic factor‐related genes. (H, I) Changes in mRNA levels of hematopoietic factors in BM‐MSCs after receiving different treatments (*n* = 3). (J) Levels of hematopoietic factors level in the bone marrow supernatant of irradiated mice on day 7 after receiving different treatments (*n* = 4). (K) Hematopoietic factors produced by BM‐MSCs which under different conditions (*n* = 4).

After co‐culturing with different gels for 48 h, RNA‐seq was conducted to examine the transcriptome profile of BM‐MSCs. The top 50 differentially expressed genes (DEGs) between groups are listed in Figure S5A. PCA analysis revealed homogeneity among replicate samples, suggesting the validity of the RNA‐seq data (Figure S5B). Moreover, different treatments dominantly affected the early events in BM‐MSCs, and most DEGs were exhibited between IR‐Con and other groups. The heatmap of cell cycle‐related genes from various treatments also suggested that there was a large fluctuation between groups (Figure 5A). We investigated the expression change of 16 cell‐cycle‐related molecules of BM‐MSCs that received different treatments. As shown in Figure 5B–E, the expression of *Checkpoint Kinase 1* (*CHEK1*), *Cell Division Cycle 6 (CDC6)*, *Cell Division Cycle 25A (CDC25A)*, *Cyclin‐Dependent Kinase 6 (CDK6)*, *Cyclin‐Dependent Kinase 2 (CDK2)*, and *Cell Division Cycle 25C (CDC25C)* were increased by Gel/Laponite@IL‐12 treatment. Furthermore, the expression of CDC25A and CDC25C on the protein level was also obviously up‐regulated by this gel treatment (Figure 5F). CDC25 phosphatases are critical regulators of cell cycle progression by phosphorylating cyclin‐dependent kinases (CDKs), and CDK2 phosphorylation plays a key role in CDK2 activation and the entrance of phase S. Therefore, we investigated the effects of Gel/Laponite@IL‐12 treatment on CDK2 phosphorylation (T14, Y15, and T160). The phosphorylation of Y15 and T14 decreased along with the up‐regulation of T160 phosphorylation in BM‐MSCs that received Gel/Laponite@IL‐12 treatment (Figure 5F). These results suggested that our hydrogel system treatment could effectively increase CDK2 activity, although it had no obvious effect on the total protein levels of CDK2 (Figure 5G). The aforementioned results provide further evidence that hydrogel treatment effectively stimulates BM‐MSCs proliferation through up‐regulation of CDC25 and activation of CDK2.

Next, we explored the impact of Gel/Laponite@IL‐12 treatment on the secretion of BM‐MSCs after irradiation. As shown in Figure 5G, the expression of hematopoietic growth factors (HGFs) changed markedly after different treatments. We analyzed the expression of HGF mRNA in different treatment groups and found that HGFs had higher expression in the Gel/Laponite@IL‐12‐treated BM‐MSCs than in the other treatments (Figure 5H,I). Furthermore, the factors that changed the most significantly at the mRNA level were verified by enzyme‐linked immunosorbent assay (ELISA). As shown in Figure 5J, the Gel/Laponite@IL‐12‐treated mice had a higher cytokine concentration than other groups. Consistent with the in vivo results, the concentration of hematopoietic factors increased significantly in the Gel/Laponite@IL‐12 group after co‐culturing for 48 h (Figure 5K). Collectively, these findings indicate that Gel/Laponite@IL‐12 treatment could potentially aid in hematopoietic recovery by enhancing the proliferation and secretion of HGFs by BM‐MSCs.

Along with the degradation of the hydrogel system, the Laponite can release magnesium ions (Mg^2+^) and strontium ions (Si^2+^), which are beneficial to promote osteogenesis and relieve radiation‐induced myelosuppression.^31^ Furthermore, our previous study demonstrated that IL‐12 activated the signal transducer and activator of transcription 3 (STAT3) signaling pathway to enhance osteogenesis.^41^ Therefore, we investigated the impact of different treatments on the osteogenic differentiation of BM‐MSCs in vitro. As shown in Figure S6A–D, Gel/Laponite@IL‐12 treatment increased alkaline phosphatase (ALP) activity and extracellular mineralization. In addition, the osteogenic differentiation of BM‐MSCs induced by this gel system treatment showed a more obvious up‐regulation trend with a prolonged co‐culture time. Furthermore, the osteogenic‐related genes fluctuated with different treatments and expressed higher levels in the Gel/Laponite@IL‐12 group (Figure S6E). The expression of osteogenesis‐specific genes also showed similar results. With the addition of Gel/Laponite@IL‐12 and the extension of co‐culture time, the expression of bone morphogenetic protein 2 (*BMP2*), *alkaline phosphatase (AKP), runt‐related transcription factor 2 (RUNX2), osteocalcin (OCN)* and collagen type I alpha 1 (*COLA1*) was significantly up‐regulated (Figure S6F–H). The expression of RUNX2 and OCN was also up‐regulated in Gel/Laponite@IL‐12‐treated BM‐MSCs on protein level at day 14 (Figure S6I). Together, these results suggested that Gel/Laponite@IL‐12 treatment enhanced osteogenic differentiation of BM‐MSCs.

### 2.5 IL12R‐PI3K/AKT signaling was involved in hydrogel‐induced BM‐MSC proliferation and promotion of HGFs secretion

Considering that the biological activities of IL‐12 depend on its receptors, we inquired whether HSCs and/or BM‐MSCs expressed the IL‐12 receptor (IL‐12R). As shown in Figure S7A, IL‐12R expression was found at very low levels in HSCs while IL‐12R expression was more abundant in BM‐MSCs and to be up‐regulated with the addition of IL‐12‐loaded gels. In BM‐MSCs, the expression of IL‐12‐related genes was found to be slightly down‐regulated in the IL‐12 unloaded group (Figure S7B). As shown in Figure S7C,D, IL‐12R expression exhibited a dramatic up‐regulation after treatment of gels loaded with IL‐12, which was confirmed by immunofluorescence and western blotting assays. These results indicated that sustained release of IL‐12 effectively activated the expression of IL‐12R, and IL‐12‐mediated radioprotection of HSCs was through indirect effects on BM‐MSCs rather than directly on HSCs.

**FIGURE 6:**
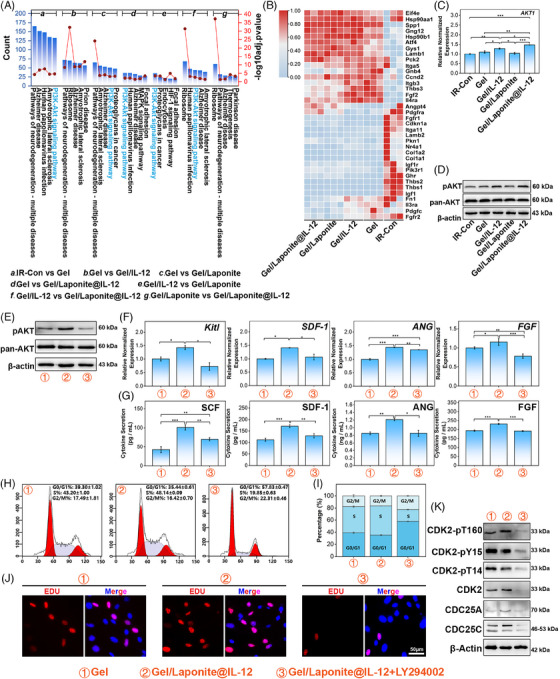
Hydrogel regulated the proliferation and secretion of bone marrow‐mesenchymal stem/stromal cells (BM‐MSCs) through the IL12R‐PI3K/AKT signaling pathway. (A) Kyoto Encyclopedia of Genes and Genomes (KEGG) analysis of BM‐MSCs cultured in different conditions (the top five pathways sorted by gene count are shown). (B) Heatmap of the expression of PI3K/AKT related genes; changes in expression of pan AKT and p‐AKT in BM‐MSCs at mRNA (C) and protein levels (D); (E) PI3K/AKT inhibitor abolished the enhancement of p‐AKT which is regulated by hydrogel. (F–K) Hematopoietic factors produced by BM‐MSCs were weakened by exposure to the PI3K/AKT inhibitor. Expression of hematopoietic factors of BM‐MSCs on mRNA levels (F) and protein levels (G). (H–K) Cell proliferation of BM‐MSCs enhanced by hydrogel was inhibited by LY294002. (H, I) Cell cycle analysis of BM‐MSCs after receiving inhibitor treatment. (J) Representative images of EdU‐positive cells in BM‐MSCs under different conditions (scale bar = 50 µm); (K) the expression of cell cycle‐related proteins in BM‐MSCs under different conditions. (*n* = 3).

The Kyoto Encyclopedia of Genes and Genomes enrichment analysis was conducted on DEGs to determine the biological functions of BM‐MSCs with various treatments. In particular, the phosphoinositide 3‐kinase/Protein Kinase B (PI3K/AKT) signaling pathway emerged in most comparisons, suggesting its crucial role in responding to the different treatments (Figure 6A). Furthermore, the heatmap of the genes of the PI3K/AKT signaling pathway (Figure 6B) indicated that the activation level of the genes expressed a similar tendency as the total DEGs in Figure S5A. To further investigate the activation of PI3K/AKT signaling among different groups, we assessed the expression of AKT, a pivotal component of the PI3K/AKT signaling pathway, at both the mRNA and protein levels (Figure 6C,D). Although pan‐AKT was not apparently different between groups of BM‐MSCs with or without hydrogel treatment, the mRNA level of *AKT1* and the phosphorylation of AKT (S473) was up‐regulated in the IL‐12‐loaded group (Figure 6C,D).

As shown in Figure 6E, LY294002, a PI3K/AKT inhibitor, abolished the up‐regulation of AKT phosphorylation caused by Gel/Laponite@IL‐12 treatment. Consistent with the result of AKT phosphorylation, the expression of hematopoietic factors at the mRNA (Figure 6F) and protein (Figure 6G) levels also reduced with the addition of the PI3K/AKT inhibitor.

The cell cycle and EdU incorporation assay were used to confirm the role of the PI3K/AKT signaling pathway in the hydrogel‐mediated proliferation of BM‐MSCs. As shown in Figure 6H,I, PI3K/AKT inhibitor treatment led to a G0/G1 phase arrest and interference with cell cycle progression, indicating that LY294002 effectively suppressed the proliferation induced by Gel/Laponite@IL‐12. In Figure 6J, the proportion of EdU‐positive cells decreased in the Gel/Laponite@IL‐12 group following the addition of the inhibitor. Furthermore, the protein expression of CDC25A and CDC25C were down‐regulated after the administration of the inhibitor. Simultaneously, total protein expression levels and phosphorylation of T160, Y15, and T14 of CDK2 decreased as a result of the use of an inhibitor (Figure 6K). Altogether, the results indicated that the PI3K/AKT signaling was involved in Gel/Laponite@IL‐12‐induced proliferation and HGF secretion of BM‐MSCs after irradiation.

## 3 DISCUSSION

As nuclear technology expands its reach across multiple domains, including military, energy, and medical fields, the urgency to investigate effective treatment approaches for ARS intensifies.^4^ A variety of aminothiol small molecules and HGFs, specifically G‐CSF, GM‐CSF, and EPO, have the ability to alleviate radiation‐induced myelosuppression.^4^, ^42^ It is worth noting that treatment with cytokines also presents major limitations, including low bioavailability and burst release of cytokines. In recent years, biomaterials have offered a novel avenue for the clinical application of cytokines, providing a new therapeutic strategy for ARS. In our study, we observed that a sustained release of IL‐12, facilitated by a nanoclay‐reinforced RGD‐Alg hydrogel, enhanced the recovery of hematopoiesis and supported the reconstitution of the hematopoietic microenvironment in a mouse model following a single intra‐BM injection. Our data demonstrated the advantages and possibilities of using an IL‐12 sustained‐release hydrogel for the treatment of H‐ARS, while also elucidating the underlying mechanisms of this hematopoietic restoration.

IL‐12 has been demonstrated to be a promising radiation medical countermeasure for the H‐ARS, as it can improve survival rates and promote hematopoietic reconstitution in the murine and nonhuman primate model of H‐ARS.^5^, ^19^, ^43^ For example, injecting mouse recombinant IL‐12 significantly improves survival rates and increases blood cell nadirs of acutely irradiated mice, accompanied by signs of hematopoietic recovery.^19^ In addition, treatment with IL‐12 also mitigates radiation damage to gastrointestinal tissues.^5^ Experimental results in nonhuman primates have also shown similar findings.^19^, ^43^ A further Phase II study suggested that the use of IL‐12 could improve blood profiles, slow down weight loss, and increase nadir counts of leukocytes, thrombocytes, and reticulocytes. Furthermore, IL‐12 enhanced immune function by improving multilineage progenitor compartments within the BM.^17^, ^44^ Regarding the mechanisms through which IL‐12 stimulates hematopoiesis, some studies have proposed that IL‐12 may restore hematopoiesis by inhibiting the P53‐dependent apoptotic pathway, while other research suggests that IL‐12 exerts its effects through interferon‐gamma (IFN‐γ).^45^, ^46^ However, IFN‐γ is a double‐edged sword in hematopoiesis: low doses of IFN‐γ can promote hematopoiesis and excessive IFN‐γ may inhibit hematopoiesis.^47^, ^48^ The toxicity of IL‐12 has been partly associated with excessive production of IFN‐γ. Thus, the side effects of IL‐12 remain a concern and have limited its application in clinical practice. For example, severe non‐hematological toxicity occurred in patients with multiple myeloma who received 250 ng/Kg intravenous IL‐12.^49^ IL‐12 can induce symptoms such as fever, abdominal pain, fatigue, and nausea when administered intravenously at 600 ng/Kg.^50^ Systemic‐to‐local administration is a highly effective strategy to reduce the side effects of most cytokines, whereas local and sustained drug release helps minimize the systemic toxicity associated with pro‐inflammatory factors. Furthermore, previous studies have documented that a combined strategy employing intra‐BM injection (IBMI) and biomaterials improves the therapeutic efficacy of co‐doped trace element calcium phosphate particles in treating osteoporosis.^51^ Therefore, we designed a biomaterial‐based delivery carrier with IBMI that could achieve local and sustained administration of IL‐12.

Alginate is usually used as a carrier for therapeutic proteins and has been shown to be safe in drug delivery applications.^52^ It can form egg‐box crosslinking in the presence of divalent metal cations, and this physical cross‐link leads to a microporous structure within the gel, which is conducive to the infiltration of tissue cells.^27^, ^30^, ^38^ However, this inner structure can induce a burst release of proteins, which partially explains why there was no significant difference between IL‐12 treatment and Gel/IL‐12 treatment in terms of mouse survival and hematopoietic repair after irradiation. However, the integration of nanoparticles has the potential to improve this undesirable characteristic of hydrogels.^27^ Laponite was selected as an incorporation particle to prevent the burst release of IL‐12 in our study. These nanoparticles engage with proteins through various forces, including hydrogen bonding, Van der Waals interactions, and electrostatic adsorption. Specifically, the negatively charged surface of the Laponite nanoclay has the ability to bind to positively charged domains on the protein, which can facilitate the sustained release of IL‐12 from the hydrogel.^31^ As a common thickener and plasticizer, Laponite can change the mechanical properties of materials.^53^ The alteration in stiffness could be attributed to the presence of free amine and carboxyl groups on the Alg base, together with the charged surface of the Laponite nanoclay.^30^ The Young's modulus of RGD‐Alg/ 1% Laponite hydrogel was ∼9 kPa, which was similar to the values of Young's modulus of BM at physiological temperature (0.25–24.7 kPa).^54^ In this study, Gel/Laponite@IL‐12 was specially crafted and utilized as a carrier to facilitate the localized and continuous release of IL‐12 over a period of 28 days.

The combined therapeutic strategy of IBMI and the sustained release IL‐12 hydrogel system could effectively improve the survival of mice under sublethal irradiation by enhancing multilineage hematopoietic reconstitution. The synergistic effect of topical and sustained‐release administration contributed to therapeutic outcomes. The excessive production of IFN‐γ accounts for the toxicity of IL‐12, and local administration or low‐dose attenuates these toxic effects.^21^, ^55^ In addition, the toxicity and the enhancement of hematologic recovery of IL‐12 were also related to the time of administration.^5^ Previous studies emphasized that a single injection of IL‐12 is sufficient to rescue radiation‐injured hematopoiesis and repeat dose regimens would lead to excessive clinical toxicity.^56^ Although a single intraosseous injection of IL‐12 (Gel/IL‐12) could mitigate radiation‐induced hematopoietic injury in mice, the Gel/Laponite@IL‐12 treated group could achieve much better clinical efficacy, including better recovery of PBCC, longer survival period, and a larger number of HSCs in this study. However, the underlying mechanism of IL‐12 leading to hematopoietic recovery remains controversial. Fardoun‐Joalland et al. reported that IL‐12 could directly enhance the cell colonies of BM mononuclear cells in vitro.^57^ In contrast, Chen et al. noted that there was no detectable expression of IL‐12R on the HSCs.^5^ Increasing evidence indicates that BM‐MSCs respond to IL‐12 stimulation, and this responsiveness is crucial for the reconstitution of the hematopoietic microenvironment and the recovery of hematopoiesis.^5^, ^41^ Our results are consistent with previous reports of no detectable IL‐12R in HSCs, but IL‐12R was highly expressed on the surface of BM‐MSCs. Furthermore, enhanced expression of IL‐12R was observed following Gel/IL‐12 or Gel/Laponite@IL‐12 treatment, and may be involved in the promotion of the proliferation and secretion function of BM‐MSCs.

The PI3K/AKT pathway plays a vital role as an intracellular signal transduction pathway, mediating various cellular responses such as cell survival, metabolism, proliferation, growth, and angiogenesis in reaction to extracellular stimuli^58^, ^59^; however, the involvement of the PI3K/AKT pathway in the proliferation and secretion of BM‐MSCs has not been reported yet. Transcriptome analyses indicated that the PI3K/Akt pathway was among the most notably upregulated response pathways observed in this study after IL‐12 treatment. AKT phosphorylation plays a critical role in the activation of the downstream pathway and cellular functions, and we found that the Gel/IL‐12 and Gel/Laponite@IL‐12 groups could enhance the phosphorylation of AKT at S473, which ultimately promoted cell cycle progression. CDK2 acts as a regulatory molecule in cell cycle progression, and studies have shown that CDK2's main phosphorylation sites, Y15, T160, and T14, affect cell cycle progression differently. Specifically, T160 promotes the cell cycle, while T14 inhibits it. In fact, our study attested that there was indeed an up‐regulation of T160 phosphorylation in Gel/Laponite@IL‐12‐treated MSCs, and Y15/T14 phosphorylation also showed opposite trends. CDC25A and CDC25C can promote cell cycle progression by activating CDK. Our study found that the addition of the Gel component could up‐regulate the expression of CDC25A and CDC25C, which may be due to the effect of RGD grafted in RGD‐Alg on cell proliferation.^60^ Furthermore, we found that Gel/Laponite@IL‐12 treatment could significantly promote cytokines secretion of BM‐MSCs and could increase the number of hematopoietic clones in the BM‐MSCs‐HSCs co‐culture system. However, the PI3K inhibitor suppressed IL‐12‐induced phosphorylation of AKT, cell cycle activation and cytokines secretion of BM‐MSCs, further confirming the underlying role of the PI3K/AKT pathway in IL‐12‐mediated functional restoration of BM microenvironment. Furthermore, degradation of Laponite or stimulation by IL‐12 triggered osteogenesis in BM‐MSCs, while osteoblasts actively regulated stem cell fate within the hematopoietic niche. This study found a better therapeutic efficacy of Gel/Laponite@IL‐12 treatment, which supports the involvement of Laponite and IL‐12 in hematopoietic reconstitution after exposure to irradiation.

In this study, we successfully developed a well‐defined IL‐12‐sustained release hydrogel system that enhanced hematopoietic recovery and improved survival rates following sublethal irradiation. We demonstrated that this hydrogel system could activate the PI3K/AKT signaling pathway via IL‐12R, and promote the proliferation and secretion of irradiated BM‐MSCs, thus accelerating hematopoietic recovery. At the same time, Laponite and IL‐12 could synergistically improve the osteogenic differentiation of BM‐MSCs, thus benefiting the restoration of the BM microenvironment.

The limitations of this study should also be acknowledged. First, our findings are primarily based on sublethal irradiation doses, and the further efficacy of the IL‐12 sustained‐release system requires additional confirmation in a mouse model subjected to lethal irradiation doses. Second, although we have demonstrated the positive effects of IL‐12 and Laponite on osteogenic differentiation in BM‐MSCs, the underlying molecular mechanisms are still incompletely understood. Finally, the long‐term effects of this treatment remain to be fully explored.

Overall, this work provides a way to sustainably release IL‐12, which not only exploits the hematopoietic repair ability of the cytokine, but also effectively avoids the toxicity of systemic administration, and offers a novel approach to alleviate radiation‐induced hematopoietic damage.

## 4 MATERIALS AND METHODS

### 4.1 Materials

The materials utilized in our study have been comprehensively documented in the Supporting Information.

### 4.2 Synthesis and characterization of RGD‐Alg

RGD‐Alg was prepared according to the methodologies outlined in the Supporting Information. Upon preparation, the product, RGD‐Alg, was frozen and preserved at −20°C for future applications. The effectiveness of the production process for RGD‐Alg was verified by XPS.

### 4.3 Preparation and characterization of RGD‐Alg/Laponite@IL‐12 hydrogel

The preparation of RGD‐Alg/Laponite@IL‐12 gel followed the methods detailed in the Supporting Information. Rheological evaluations were performed using a Discovery II rheometer (TA Instruments). Compression tests determined the compression modulus of the hydrogels, while injection tests evaluated their shear‐thinning characteristics. The degradation rate of the hydrogels was calculated based on the residual dry mass after incubation in the medium for various time points. The internal hydrogel structure was visualized through SEM (FEI Nova 400 Nano SEM) of lyophilized cross‐sections. The controlled‐release capabilities of the encapsulated IL‐12 were measured using ELISA. Detailed methodologies for these experiments are provided in the Supporting Information.

### 4.4 Cell culture, isolation, and irradiation in vitro

A complete culture medium for C57BL/6 mouse BM‐MSCs and BM‐MSCs was purchased from Cyagen. Cells were passaged when they reached 70%–80% confluence and cells from the 3rd to 5th passages were utilized for subsequent experiments. BM cells were extracted from C57BL/6 mice according to the manufacturer's guidelines using a mouse BM mononuclear cell isolation kit. A total of 1 × 10^5^ cells were seeded in 24‐well plates using 1 mL of RPMI‐1640 medium enriched with 10% v/v fetal bovine serum, 1% v/v penicillin/streptomycin, and 50 µM β‐mercaptoethanol. For irradiation experiments, cells underwent hydrogel treatment immediately after exposure to 5 Gy IR (Co60).

### 4.5 In vivo characterization of hematopoietic system regeneration in a radiated mouse model

For total body irradiation (TBI), 6–8‐week‐old male C57BL/6 mice were irradiated with 5 Gy or 7 Gy using a Co60 irradiator. To test the radiation regeneration effect of RGD‐Alg/Laponite@IL‐12, the irradiated mice were injected intra‐marrow with the hydrogel system (0.5 mL/kg) within 1 h after TBI, and given an equal volume of normal saline to be used as control. The PBCCs of mice were analyzed by the MAXCOT Multispecies Hematology System (MAXCOT) at various time points after injection. For BM hematopoietic regeneration and osteogenesis assays, mouse femurs were preserved in 4% paraformaldehyde, then decalcified and embedded in paraffin for the preparation of 5 µm microsections. Subsequently, these sections were stained with hematoxylin and eosin (HE). Slide imaging was performed using an upright metallurgical microscope (Olympus).

### 4.6 Statistical analysis

Unless otherwise specified, the data presented represent the mean ± standard deviation calculated from a minimum of three distinct experiments. Statistical evaluation was conducted using Origin 2023 software. To analyze multiple groups, a one‐way analysis of variance was used, paired with a Tukey test for parametric data or a Kruskal‐Wallis test for nonparametric data. Statistical significance was established at a *p*‐value of < 0.05 and is represented as follows: *, *p* < 0.05; **, *p* < 0.01; ***, *p* < 0.001; ****, *p* < 0.0001; ns indicates non‐significance.

## AUTHOR CONTRIBUTIONS

*Qian Ran, Zhongjun Li, Jigang Dai, and Li Chen*: contributed to the conception and design of the projects. *Chuanchuan Lin and Yang Xiang*: performed most of the experiments, analyzed the data, and drafted the manuscript. *Yangyang Zhang, Nanxi Chen, Weiwei Zhang, Lanyue Hu, Jianxin Chen, Ya Luo, Xueying Wang, Yanni Xiao, Qing Zhang, and Xi Ran*: participated in the experimental work. *Zhenxing Yang*: edited the manuscript and participated in the data analysis. All authors have read and approved the article.

## CONFLICT OF INTEREST STATEMENT

The authors declare no conflict of interest.

## ETHICS STATEMENT

All animal experiments were approved by the Laboratory Animal Welfare and Ethics Committee of the Army Medical University (AMUWE20234503).

## Supporting information

Supporting Information

## Data Availability

The data that support the findings of this study are openly available in GEO with accession number GSE267635 upon reasonable request.
