# Supplementary material for: Interleukin‐12 sustained release system promotes hematopoietic recovery after radiation injury
Source: MedComm (2020). 2024 Sep 12;5(9):e704. doi: 10.1002/mco2.704 (PMC11391269; doi:10.1002/mco2.704)
Supplement: Supplementary file 1 — Supporting Information [file MCO2-5-e704-s001.docx]

Supplementary Materials for

Interleukin-12 sustained release system promotes hematopoietic recovery after radiation injury

Chuanchuan Lin ^1,2,#^, Yang Xiang ^1,2, #^, Yangyang Zhang ^1,2^, Zhenxin Yang ^1,2^, Nanxi Chen ^1,2^, Weiwei Zhang ^1,2^, Lanyue Hu ^1,2^, Jianxin Chen ^1,2^, Ya Luo ^1,2^, Xueying Wang ^1,2^, Yanni Xiao ^1,4^, Qing Zhang ^5^, Xi Ran ^6^, Li Chen ^1,2,^*, Jigang Dai ^2,3,^*, Zhongjun Li ^1,2,^*, Qian Ran ^1,2,^*

Correspondence to: chenli200401@163.com, daijigang@tmmu.edu.cn, zhongjunli@tmmu.edu.cn, louise-r-q@163.com

**This PDF file includes:**

Materials and Methods

Figures S1 to S7

Tables S1 to S2

Captions for Figures S1 to S7

Materials and Methods

Materials

Sodium alginate, 2-(N-Morpholino) ethanesulfonic acid (MES), 1-(3-dimethylaminopropyl)-3-ethylcarbodiimide hydrochloride (EDC), N-hydroxysuccinimide (NHS) and calcium sulfate (CaSO_4_) were purchased from Aladdin Industrial Co. (Shanghai, China). GGGGRGDSP peptide was synthesized by APeptide Co. (Shanghai, China). Fluorescein diacetate (FDA), propidium iodide (PI), Rhodamine-conjugated phalloidin, and DAPI were supplied by Sigma Chemical Co. (St. Louis, MO, USA). LY249002 was purchased from Selleck Co. (Shanghai, China). The Cell Counting Kit- 8 (CCK8) was obtained from APE × BIO Technology LLC (Houston, TX, USA). A total RNA extraction kit was provided by Tiangen (Beijing, China). PrimeScriptTM RT reagent kit with gDNA eraser and TB GreenTM fast qPCR kit were obtained from Takara (Dalian, China). Trizol and all primers were obtained from Invitrogen Co. (CA, USA). The cell cycle analysis kit was purchased from BD Biosciences (NJ, USA). EdU cell proliferation kit with Alexa Fluor 549, BCIP/NBT alkaline phosphatase color development kit, Alkaline phosphatase assay kit, and RIPA lysis buffer were obtained from Beyotime (Chengdu, China). Mouse MSCs medium was purchased from Cyagen Biosciences Co. (Guangzhou, China). ELISA kits were all purchased from Shanghai Jianglai Industrial Limited by Share Ltd., (Shanghai, China). 1640 medium, fetal bovine serum (FBS), and trypsin-EDTA solution were obtained from Gibco (NY., USA). C57/BL6 mouse was provided by Beijing Vital River Laboratory Animal Technology Co., Ltd. (Beijing, China).

Synthesis of RGD-Alg

The low molecular weight alginate was produced by irradiating high molecular weight sodium alginate using Co60 radiation at 5 Mrad, similar to methods described in our previous study. Initially, low molecular weight alginate underwent grafting with RGD. Specifically, a MES buffer solution with a pH of 6.5 containing 2% w/v alginate, 2.5 mM EDC, and 2.5 mM NHS was stirred continuously for 30 minutes. Subsequently, 1500 μM of GGGGRGDSP was introduced, and stirring continued for 24 h. The resulting product was then dialyzed in deionized water using a dialysis bag with a molecular weight cutoff of 3.5 kDa for 3 days, with frequent water changes. Finally, the product was dried using a vacuum freeze dryer.

Preparation of the RGD-Alg/Laponite@IL-12 hydrogel

The precursor hydrogel solution was formulated by blending the RGD-Alg solution with various concentrations of Laponite solution. Specifically, Laponite dispersions were prepared at concentrations of 0, 1.25, and 2.5 wt% in deionized water and vigorously stirred for 30 minutes to ensure stability. The RGD-Alg solution (5% w/v) was then introduced into the Laponite dispersions at a 1:1 ratio, followed by vortex mixing for 10 minutes to ensure homogeneity. For IL-12 incorporation, 5 μg/mL of IL-12 was mixed with the Laponite dispersions for 15 minutes prior to combining with the alginate solutions. Finally, the precursor hydrogel solution was combined with a CaSO_4_ slurry to facilitate physical crosslinking, resulting in a final hydrogel composition of 2% w/v RGD-Alg, 0.5% or 1% w/v Laponite, and 20 mM CaSO_4_.

Physicochemical characterization and IL-12 release properties of hydrogel

Rheological measurements were performed using a Discovery II rheometer (TA Instruments, U.S.A) equipped with a parallel plate (diameter= 40 mm, gap= 50 μm). At least triplicates were carried out for each rheological measurement. The compression modulus of hydrogels was measured with compression tests. In brief, hydrogel disks (diameter= 8 mm, height= 4 mm) were placed on an Instron 5567 (MA. USA) platform, and the gel was compressed to 15% strain. The deformation rate was 1 mm/min, and the initial elastic modulus was the slope of the first 5-15% strain stress-strain curves. Injection tests were used to determine the shear-thinning properties of the hydrogels. The RGD-Alg/Laponite@IL-12 hydrogel precursor solution was quickly mixed with 20 mM CaSO_4_ and filled into a 1 mL syringe. A 26G needle was used to inject the hydrogel into the saline and a clean slide surface, and the cross-linking of the hydrogel was observed.

The degradation rate of hydrogels was obtained by calculating the residual dry mass of hydrogel after different incubation times in the medium. The internal structure of the hydrogel was observed by scanning electron microscopy (FEI Nova 400 Nano SEM, Holland) of the cross-section of lyophilized samples. The controlled-release properties of loaded IL-12 were assessed by ELISA. The Laponite which loaded IL-12 was dispersed in RGD-Alg hydrogel, and the hydrogel system was immersed in sterilized PBS in a 37 ^o^C incubator. Next, the supernatants from the incubation solutions were collected and frozen stored at -80 ^o^C at day 0, 1, 3, 7, 14, and 28. The IL-12 was measured using ELISA kits following the manufacturer instructions.

Cell viability assay

After co-culturing with different hydrogels for 48 h, the cell activity of BM-MSCs or bone marrow cells was assessed by FDA/PI staining. In brief, the staining solution (10 μg/mL FDA and 10 μg/mL PI) was added into each well and was allowed to incubate at 37 ^o^C for 10 min, before removing the culture medium. Then live/ dead cells of samples were imaged and counted by immunofluorescence microscopy (Olympus, Japan). The cell viability was measured via CCK-8 analysis. CCK-8 solution was added into each well on a 1:10 v/v medium, after incubating for 2 h, the OD values were measured at 450 nm (Molecular Devices SpectraMax).

Immunofluorescence analysis

The BM-MSCs were cultured with a hydrogel system for 48 h, and then the medium was removed from wells and the cells were washed twice with PBS. Next, the cells were fixed with 4% paraformaldehyde solution for 10 min, and 0.1% Triton X-100 was added for another 10 min. Next, the samples were incubated with 5% BSA solution for elimination of nonspecific binding. For the staining of the cytoskeleton, cells were incubated with rhodamine-phalloidin solution at RT for 2 h. For the staining of IL-12R, rabbit anti-IL-12R antibody solution was added and incubated overnight, the primary antibody solution was removed and washed twice with PBS. Finally, the cells were incubated with Alexa Fluor 488-goat anti-rabbit IgG solution at RT for 1 h. After staining with DAPI solution for 10 min, the cells were imaged by immunofluorescence microscopy (Zeiss LSM 880, Germany).

Flow cytometry

For marrow LSK cells (lineage-negative (Lin-) / Sca1+ / c-Kit+), HSCs (Lin- / Sca1+ / c-Kit+ / CD150+ / CD48-) and BM-MSCs (CD45- / Ter119- / CD31- / CD51+ / CD140α+) analysis, whole bone marrow cells were collected by flushing the isolated femurs and tibias with PBS. Single cells were obtained by digesting bone and marrow contents with collagenase, and the cells were resuspended and stained with cytometry antibodies for 30 min. Finally, the cells were detected and counted by CytoFLEX LX (Beckman, USA). The primary antibodies used in flow cytometry are listed in Table S1. The cell cycle analysis was performed according to the manufacturer’s instructions (BD). After the cells were treated, the cells were collected and washed with PBS and fixed with 70% ethanol. Next, cells were resuspended in PBS containing PI and RNaseA, and the DNA content was measured by flow cytometry (Gallios, BeckmanCoulter).

ELISA

The contents of stromal cell-derived factor 1 (SDF-1), stem cell factor (SCF), Angiopoietin (ANG), and fibroblast growth factor (FGF)in the bone marrow and cell culture medium were obtained by ELISA. Briefly, the mouse femurs from different treatment groups were isolated, and the marrow supernatant was obtained after high-speed centrifugation at 12000 rpm for 5 min. The conditioned media of cells in various groups were collected and centrifugated at 3000 rpm for 5 min to remove cell debris. Next, the concentration of related cytokines was detected according to the ELISA kit instructions.

Western blotting

The related protein expression of cells under different treatment conditions was performed by western blotting. After co-culture of BM-MSCs with hydrogel for 48 h, the cells were collected and lysed by RIPA buffer which contained 1% PMSF protein inhibitor. The protein samples were separated by polyacrylamide gel electrophoresis and then transferred to the 0.22 μm PVDF membrane. After blocking, the PVDF membrane was incubated with primary antibody overnight at 4°C. Following HRP-conjugated secondary antibody incubation, the protein expression was visualized by the ECL system and normalized to β-actin. The primary antibodies used in western blotting are listed in Supplementary Table S1.

qRT-PCR and RNA-seq

qRT-PCR was employed to quantify the mRNA expression of cells. First, BM-MSCs under different treatment conditions were collected and lysed by Trizol, and a total RNA extraction kit was used to extract the total RNA of BM-MSCs. After total RNA was reversed into cDNA, the relative expression of mRNA was detected by qPCR kit. Gene expression was quantified using the ΔΔCt method and fold change was calculated using formula 2^-ΔΔCt^. Values for the genes of interest were normalized with the β-actin gene. The primers (Invitrogen, CA., USA) used in the qRT-PCR analysis are listed in Supplementary Table S2.

After total RNA extract as described above, all samples were submitted to Knorigene Technologies Co., Ltd (Chongqing, China) on a HiSeq 2000 platform. Kyoto Encyclopedia of Genes and Genomes (KEGG) analysis was carried out using DAVID to identify enriched biological functional signaling pathways. All sequencing data are deposited in the NCBI GEO database under accession number: GSE267635.

Colony-forming cell assay

The bone marrow nucleated cells from non-irradiation C57/BL6 mice were isolated as indicated above. After cell counting, viable cells were mixed with expansion and culture of hematopoietic cells serum-free medium (STEMCELL, 09600) and plated at 10 cm cell culture dish. After 72 h of culture, the bone marrow nucleated cells were collected and suspended in StemSpan SFEM II, and plated in SmartDish (STEMCELL, 27370) at 1×10^5^ cells per well in triplicate for different groups, respectively. A 0.4 μm Transwell chamber was inserted into every well and the 5 Gy irradiated-BM-MSCs with or without gels were placed in the upper chamber. The total number of bone marrow-derived colony-forming unit (CFU), burst-forming unit-erythroid (BFU-E), colony-forming unit-granulocyte and macrophage (CFU-GM), and colony-forming unit-granulocyte, erythrocyte, monocyte and megakaryocyte (CFU-GEMM) were counted via microscope after 14 days of co-culture.

EdU incorporation assay

The incorporation ratio of EdU in BM-MSCs was used to evaluate cell proliferation. After receiving different treatments for 48 h, the EdU was added to the culture medium at a final concentration of 10 μM. After 2 h of co-culture, cells were fixed and EdU was tracked according to the kit instructions.


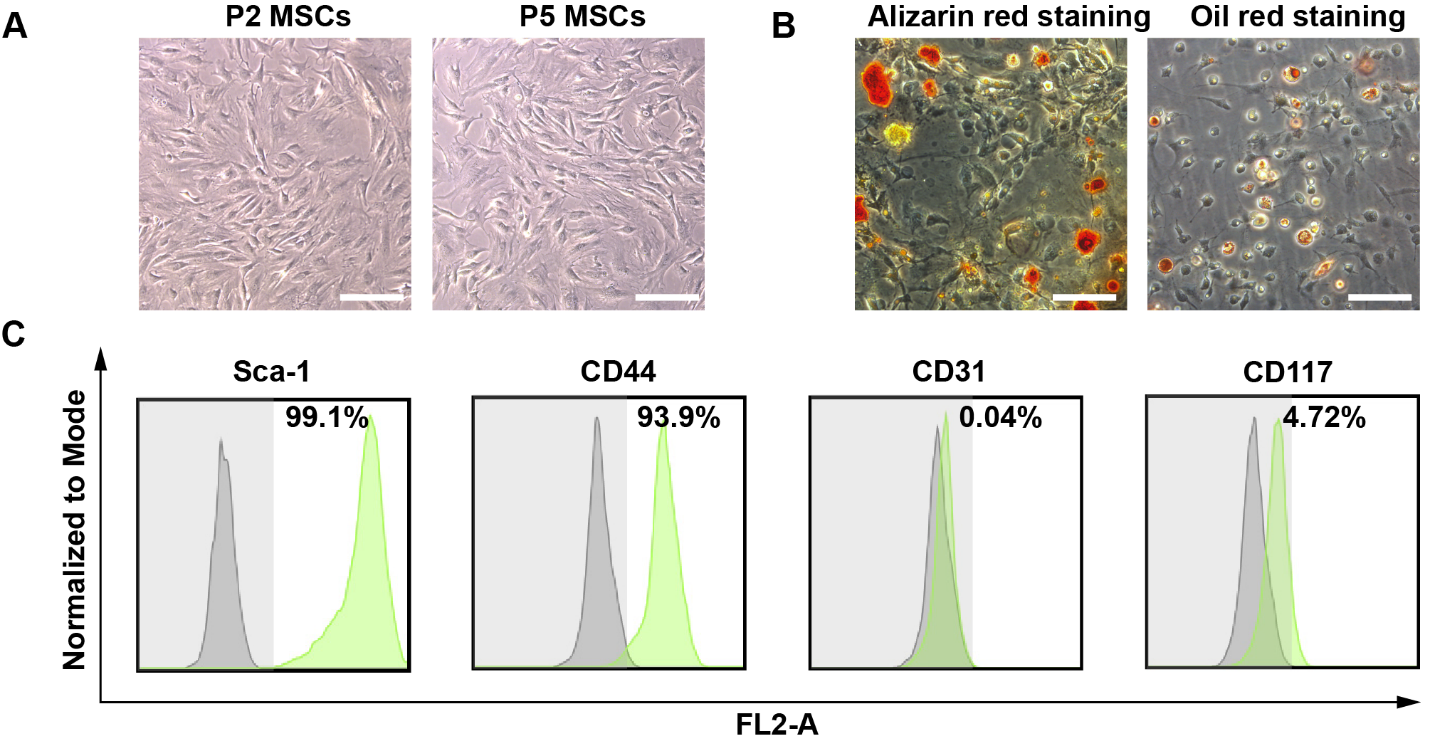


FIGURE S1 (A) Representative images of BM-MSCs morphology; (B) representative images of osteogenic and adipogenic differentiation of BM-MSCs; (C) flow cytometric analysis of BM-MSCs surface marker expression.


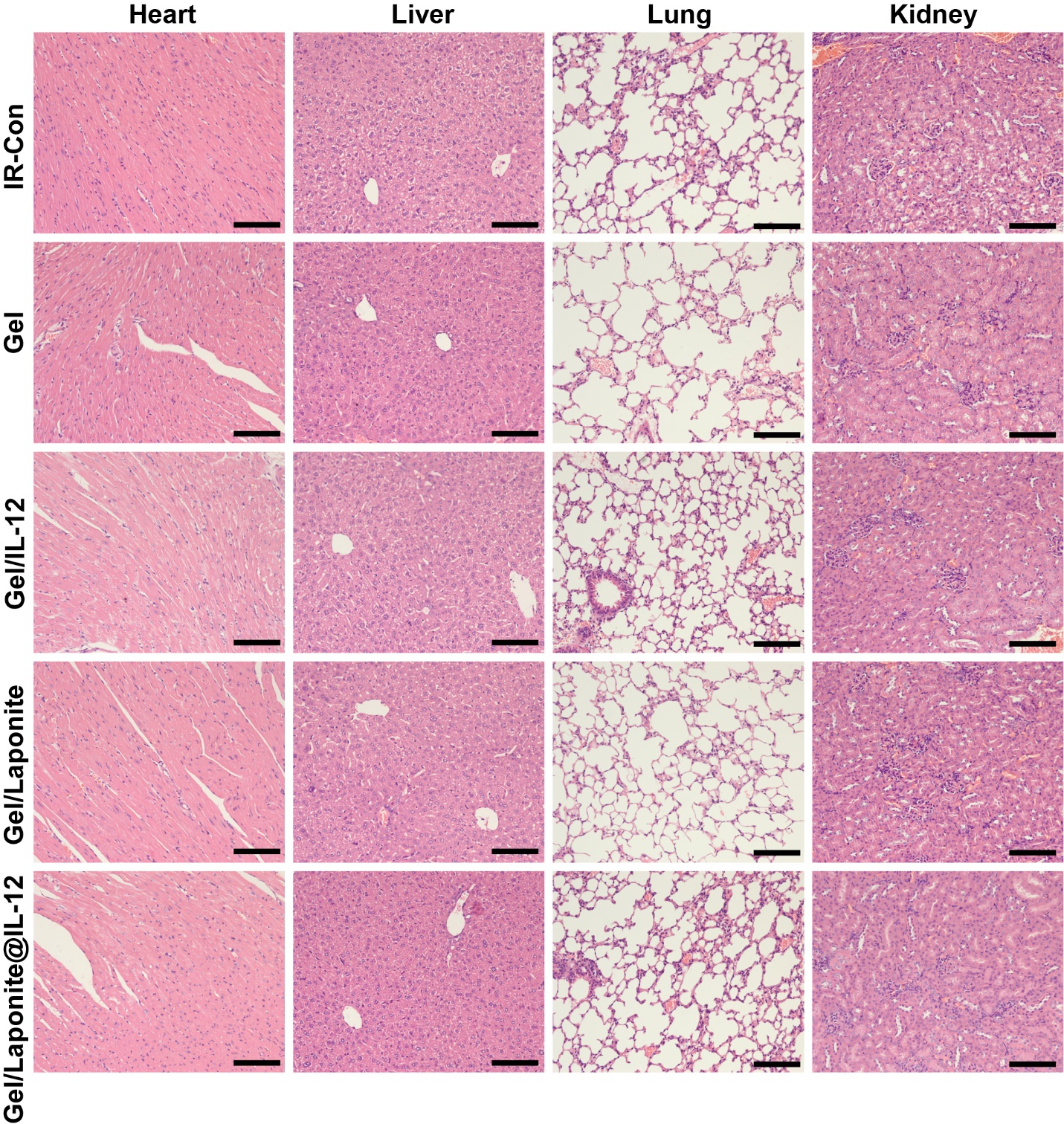


**FIGURE S2** H&E staining of major organs of mice after irradiation and different treatments on day 7.


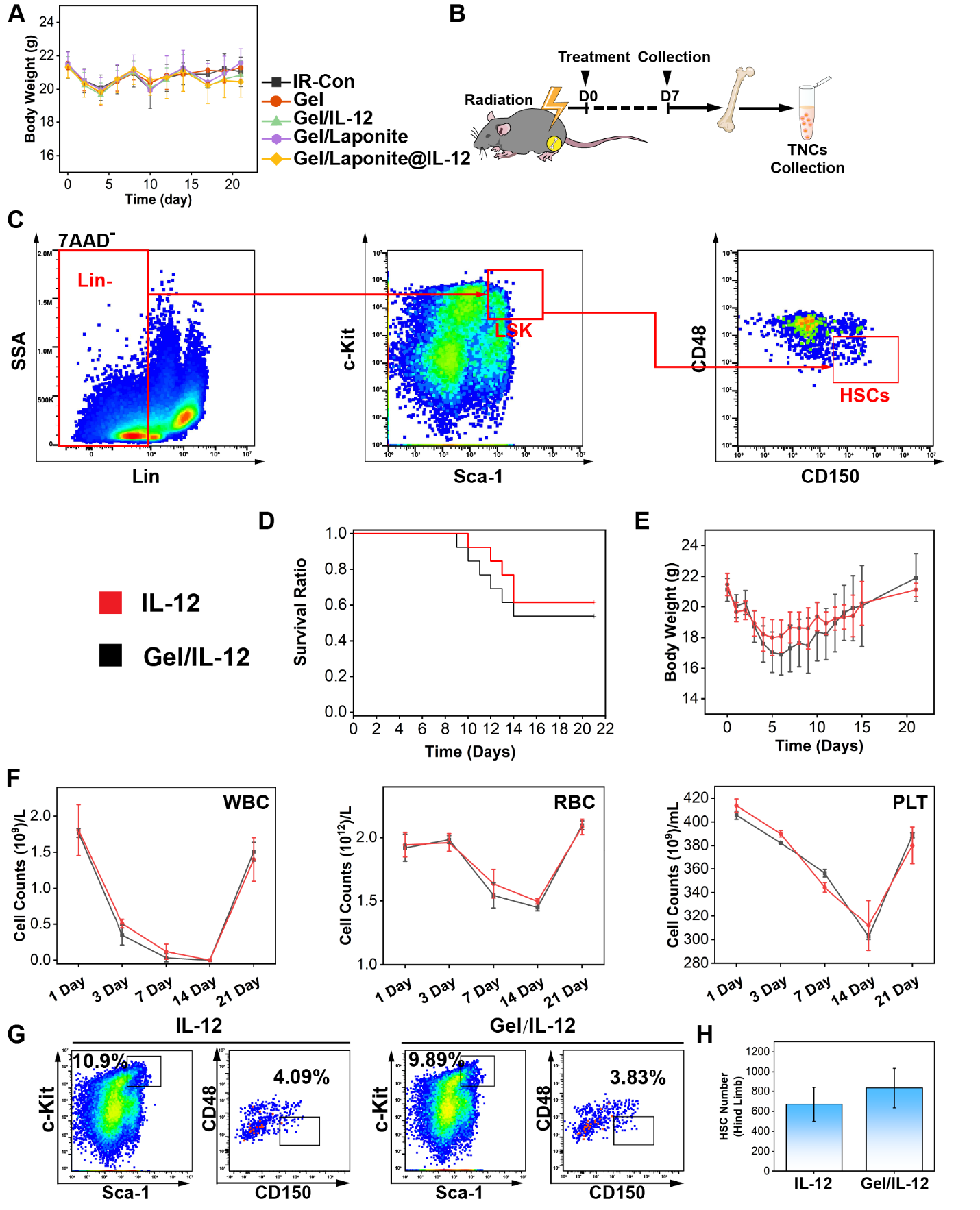


**FIGURE S3** (A) Body weights of mice with different treatments after receiving a 5 Gy irradiation dose; (B) Schematic diagram of the flow cytometry experimental process. (C) Representative flow cytometry analysis of HSCs. The effects of IL-12 and Gel/IL-12 treatment on the survival ratio (D), body weight (E), PBCC (F) and HSCs recovery (G-H) of mice after 7 Gy irradiation (n= 3).

**
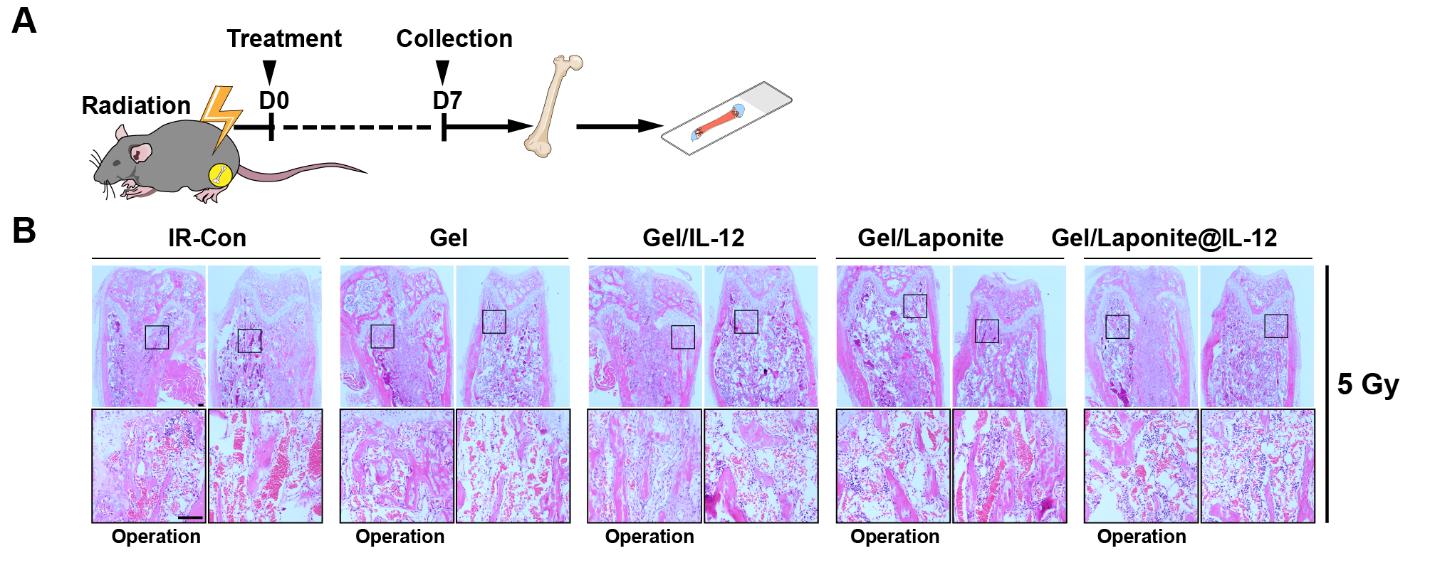
**

**FIGURE S4** (A). Schematic diagram of the experimental process; (B) H&E staining image of mouse bone marrow slides on day 7 after irradiation and different treatments (scale bar = 100 μm).


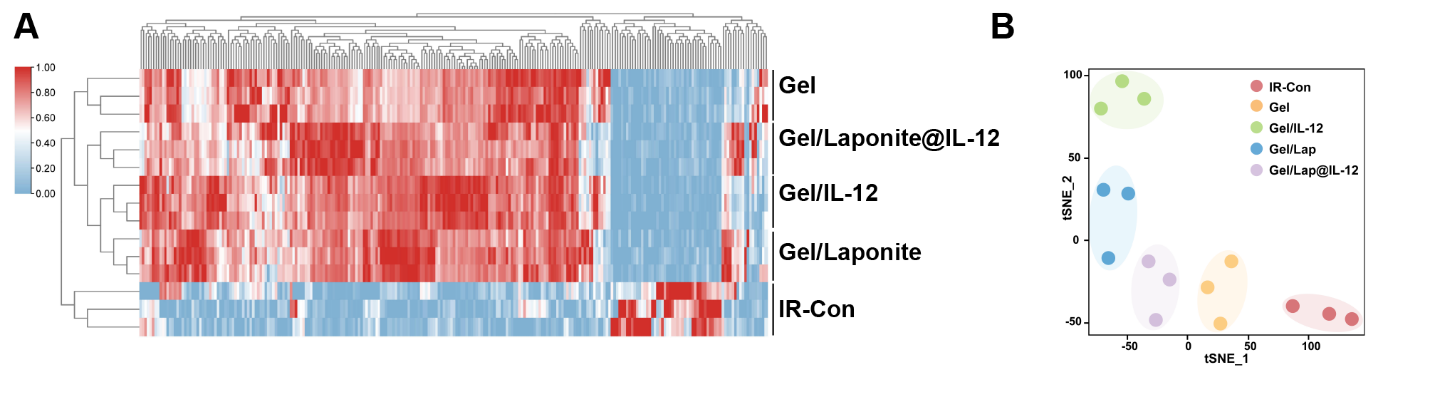


**FIGURE S5** (A) The hierarchical clustering analysis of genes up-regulated (red) and down-regulated (blue); (B) the PCA analysis of different groups.


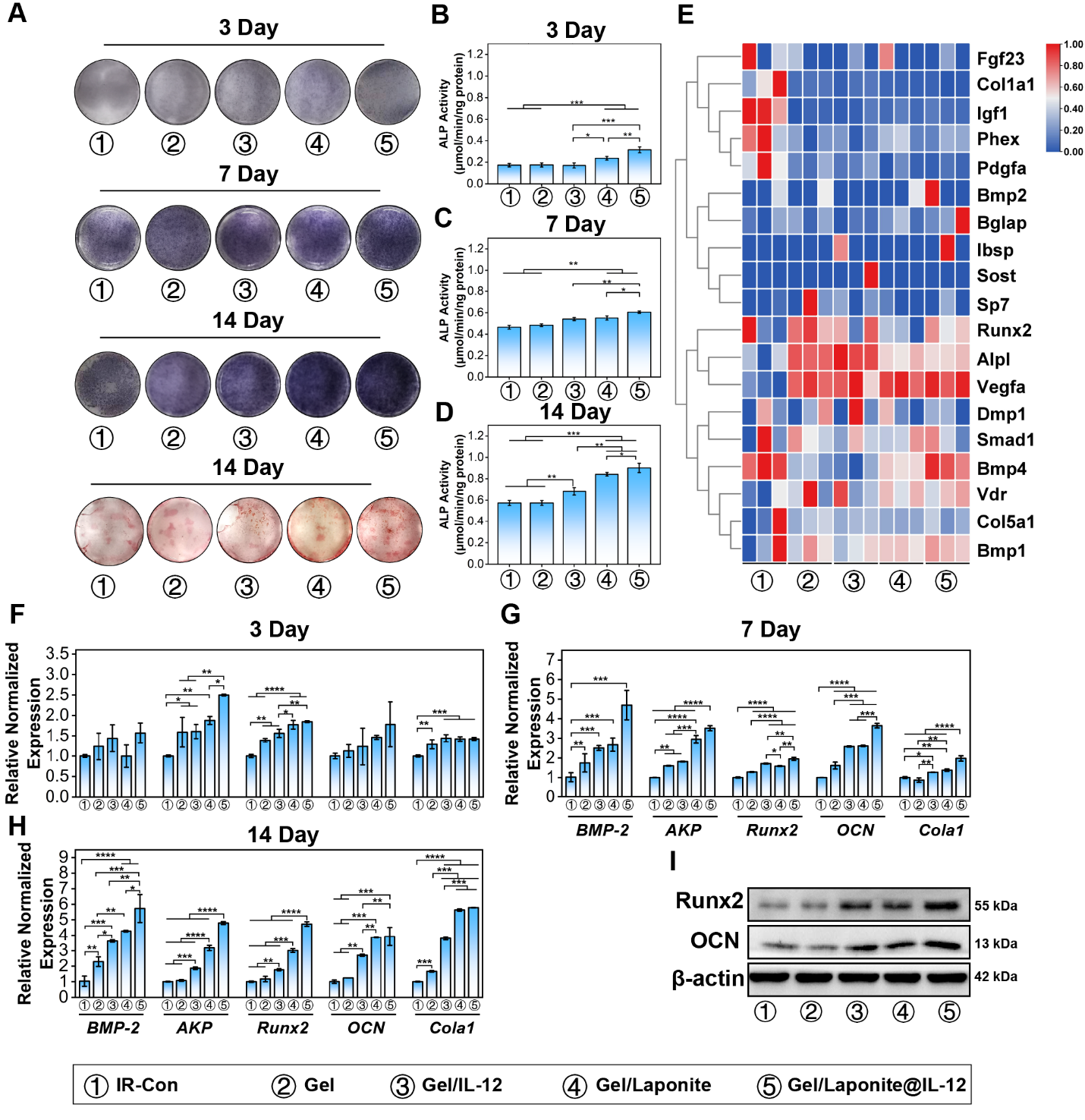


**FIGURE S6** Gel/Laponite@IL-12 enhanced hematopoiesis in irradiated-microenvironment by inducing bone formation and osteogenic of BM-MSCs. (A) Representative images of ALP and ARS staining of BM-MSCs driven by different treatment. (B-D) AKP activity of BM-MSCs after co-culturing with different gels; (E) the heatmap of the expression of osteogenic related genes; (F-H) Relative osteogenic-specific mRNA expression of BM-MSCs cultured with different gels; (I) Western blotting analysis of RUNX2 and OCN in BM-MSCs after receiving different treatment at day 14.


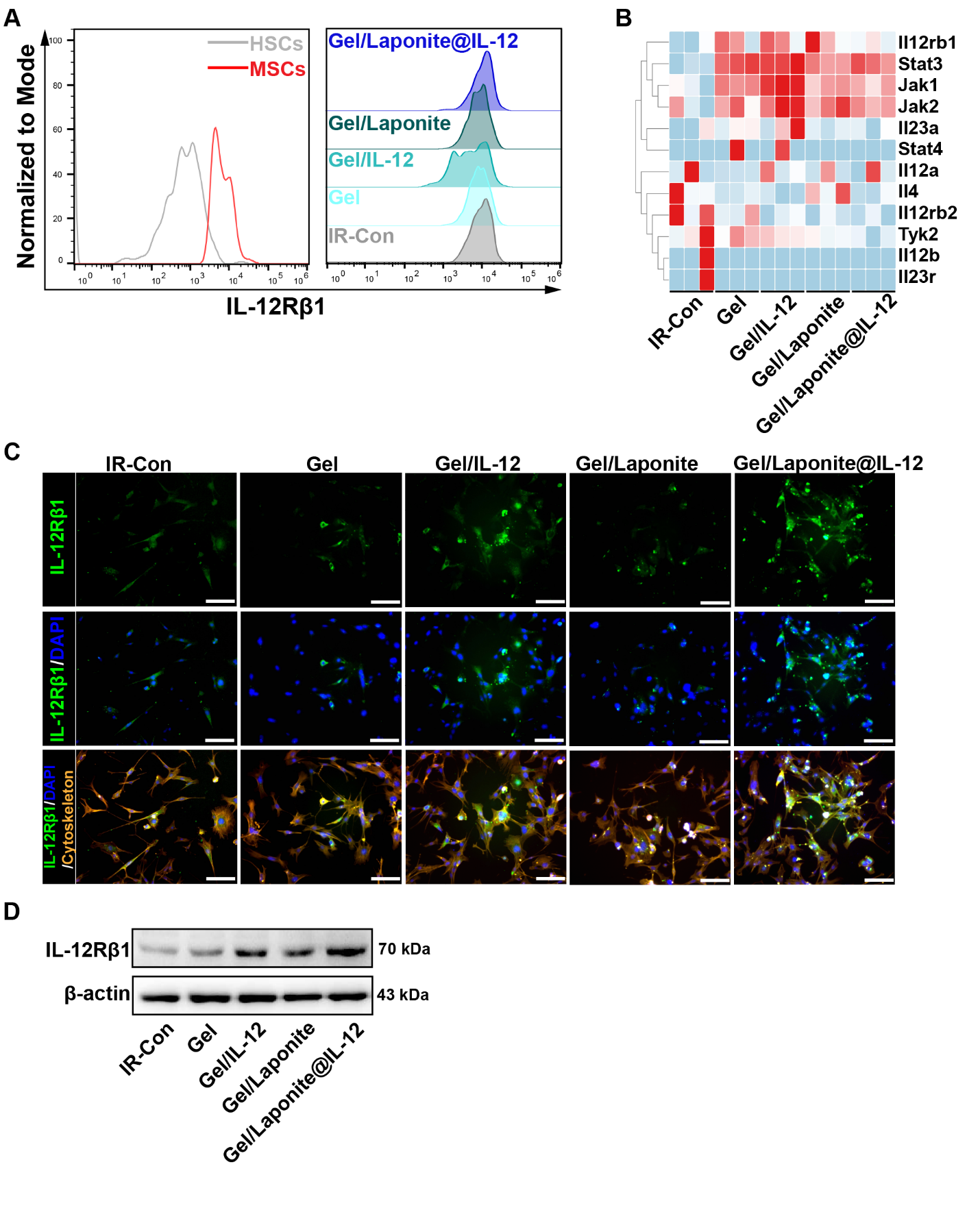


**FIGURE S7** (A) The expression level of IL12R on HSCs and BM-MSCs; (B) heatmap of the expression of IL12 related genes; (C) the representation images of IL12Rβ1 expression in BM-MSCs after receiving different treatment (scale bar= 100 μm); (D) the expression changing of IL12Rβ1 in BM-MSCs at protein levels.

Table S1.

| **Reagent or resourse** | **Source** | **Dilution** |
| --- | --- | --- |
| PE-Cy7-PDGFRα | Biolegend | 1: 50 |
| Alexa Flour 700-CD45 | Biolegend | 1: 100 |
| PE-CD51 | Biolegend | 1: 20 |
| Pacific Blue-CD31 | Biolegend | 1: 50 |
| APC-Cy7-Ter119 | Biolegend | 1: 100 |
| FITC-Lin | Biolegend | 1: 10 |
| PE-Cy7-Sca | Biolegend | 1: 100 |
| APC-Cy7-c-Kit | Biolegend | 1: 100 |
| Pacific Blue-CD48 | Biolegend | 1: 100 |
| APC-CD150 | Biolegend | 1: 100 |
| IL-12 receptor | BD | 1: 100 |
| Rabbit anti-Akt | Abcam | 1: 1000 |
| Rabbit anti-pAkt | Abcam | 1: 2000 |
| Rabbit anti-β-Actin | Proteintech | 1: 10000 |
| Rabbit anti-CDK2-pT160 | CST | 1: 1000 |
| Rabbit anti-CDK2-pY15 | Abcam | 1: 1000 |
| Rabbit anti-CDK2-pT14 | Abcam | 1: 1000 |
| Rabbit anti-CDK2 | Abcam | 1: 1000 |
| Rabbit anti-CDC25A | CST | 1: 1000 |
| Mouse anti-CDC25C | Proteintech | 1: 1000 |
| Rabbit anti-Runx2 | Proteintech | 1: 1000 |
| Rabbit anti-OCN | Abcam | 1: 1000 |

Table S2.

| **Gene** | **Forward Primer 5’-3’** | **Reverse Primer 5’-3’** |
| --- | --- | --- |
| *BMP-2* | TGTACCGCAGGCACTCAG | GTTCCTCCACGGCTTCTT |
| *ALP* | GGTAGATTACGCTCACAACAA | AGGCATACGCCATCACAT |
| *RUNX2* | ACAGTTATCAAGGGAATAGAGG | AGGGAGGACAGAGGGAAA |
| *OCN* | GCAGGAGGGCAATAAGGT | CGTAGATGCGTTTGTAGGC |
| *COLA1* | AGACTGGAAACATCGGACAT | CACAAAGACAAGAACGAGGTAG |
| *β-actin* | TTCTTTGCAGCTCCTTCG | TTCTGACCCATTCCCACC |
| *SDF-1* | CTGCTCTGGCGCTTTGTAAC | GAGCAGGGCCTTATGAAGCA |
| *Thpo* | GGACAGTTGGAACCCTCCTG | ATCCAGAAGTCCTGGGTTCCT |
| *Kitl* | AGTTCAGTCATAGATTGGAGTTTGC | ACCTGTATCAAAAGGGTCGGG |
| *ANG* | TCCCAACAGGAAGGAAGGAGT | ATCACAACCAGACCCAGCAC |
| *ANGPT1* | GGGCACACTCATGCATTCCT | GCGTCAGCTGCGAGTACATA |
| *FGF* | ACAGCCCAGCAGTTATCACC | TTTCTCTCCTCCTCCTCCCG |
| *CSF3* | CAGCCCAGATCACCCAGAATC | GCTGCAGGGCCATTAGCTTC |
| *DLL4* | CCAGCAACCCCTGTCGAAAT | ACAGTGCTGGCCATAGTAGC |
| *PTN* | AGTTTCTCATGAGTGCAGCCC | GGTTTCTCTTTCTTCCCGGC |
| *SLIT2* | TTTTCCAGGGGTCACACTTGA | TCTGGCTGTGGGAATTTTCT |
| *PCNA* | AGATGCCGTCGGGTGAATTT | TGTTCCCATTGCCAAGCTCT |
| *CHEK* | AAAGGACTGCTTGTCGCTGT | GGCATCCCTATGTCTGGCTC |
| *CDC7* | ATCCTTCTGTTGGCACCCTG | CTCCTCCATCTGAGTCCCCA |
| *CCNE2* | TCCGAGATCTAAGAGCCACC | TGTAAACGGCTACTGCGTCT |
| *CDC6* | TCTGCAAGACTTCAAGAAGGAAG | AACACGATCATGGGGCCTTT |
| *CDKN2D* | GCAAGGAAAGGAGGGAGGTC | GTCTTGCCAAAGCGGTTCAG |
| *TGFβ1* | ACTGGAGTTGTACGGCAGTG | GGGGCTGATCCCGTTGATTT |
| *CDC25A* | GCCACTTTGTCCGATGAGGA | GATCGGCCTTAGGGTCAGAG |
| *CDC25B* | ACGCTTCCGATCCTTACCAG | GGTCTCTGGAAGCGCACATT |
| *CDC25C* | AAGTCCTGAGCTTGCCTGAC | CTTGCAGGTGGGATAGGTCC |
| *CDK1* | ACGGCTTGGATTTGCTCTCA | ACGATCTTCCCCTACGACCA |
| *CDK2* | AGCTCTCCTTGCGTTCCATC | ACGTGCCCTCTCCAATCTTC |
| *CDK4* | GTCTATGGTCTGGCCCGAAG | ATCCATCAGCCTTACGCTCG |
| *CDK6* | CCTCTCCTTCGTGAAGACTGC | CCTCCGCAGCGATTACATAGT |
| *E2F1* | GACTGCCTTGCCTGTCTGTT | GTGCACTAAGCAAGCACCAG |
| *MYC* | CGACTACGACTCCGTACAGC | GTAGCGACCGCAACATAGGA |
